# Diarrheal microbiota-derived extracellular vesicles drive intestinal homeostasis dysfunction via miR-125b/NF-κB-mediated macrophage polarization

**DOI:** 10.1080/19490976.2025.2541036

**Published:** 2025-07-30

**Authors:** Mengzhen Song, Wenjun Zhou, Jinping Fan, Chenhao Jia, Wen Xiong, Hong Wei, Shiyu Tao

**Affiliations:** College of Animal Sciences and Technology, Huazhong Agricultural University, Wuhan City, Hubei, China

**Keywords:** Extracellular vesicles, miR-125b, macrophage polarization, intestinal inflammatory injury, diarrhea

## Abstract

Gut microbiota-derived extracellular vesicles (EVs) are emerging mediators of microbiota-host crosstalk, but their roles in diarrheal diseases remain poorly understood. Here, we revealed that EVs isolated from diarrheal donors’ intestinal microbiota trigger pro-inflammatory macrophage polarization and compromise intestinal epithelial barrier integrity in both conventional and pseudo-germ-free mice, demonstrating their intrinsic pathogenicity independent of viable microbes. miRNAs sequencing analysis identified miR-125b as a highly enriched miRNA in diarrheal microbiota-derived EVs, which directly targets the 3’ untranslated region of NF-κBIA, leading to its degradation and subsequent activation of the NF-κB signaling pathway. This molecular cascade drives macrophages toward a pro-inflammatory phenotype characterized by elevated TNF-α and IL-1β secretion, ultimately disrupting tight junction proteins (ZO-1, Occludin) and increasing intestinal permeability. Strikingly, adoptive transfer of primary macrophages pre-exposed to miR-125b recapitulated barrier dysfunction in recipient mice. Our study uncovers a tripartite axis linking diarrheal microbiota-derived EVs, pro-inflammatory macrophage polarization via EVs-miR-125b, and intestinal homeostasis breakdown, highlighting the underappreciated role of EVs-borne miRNAs in reshaping host immunity. These findings position EVs as both biomarkers and potential targets for further exploration for diarrhea-related inflammatory gut disorders, offering a nanotechnology-inspired strategy to modulate EVs-mediated interkingdom communication in microbiome-associated diseases.

## GRAPHICAL ABSTRACT

**Figure:**
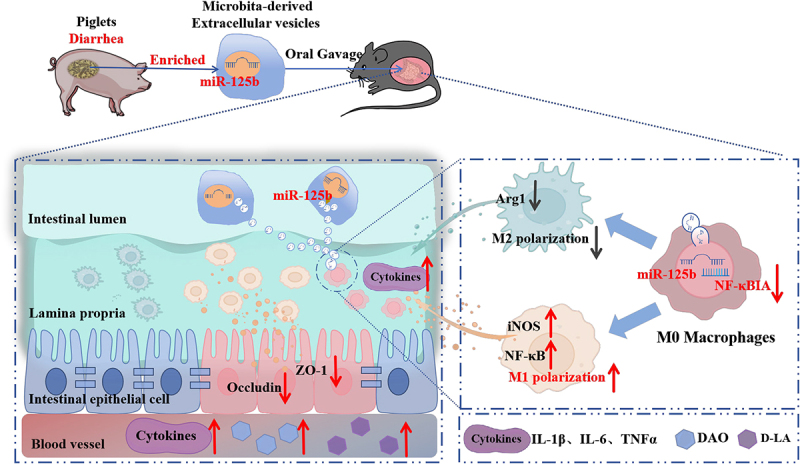

## Introduction

Diarrheal diseases represent a persistent global health challenge affecting both humans and livestock.^1,2^ Beyond their high mortality rates, these disorders are characterized by their capacity to induce intestinal inflammation and sustained impairment of gut barrier function.^3,4^ The intricate etiology and ambiguous pathological mechanisms underlying diarrheal diseases pose substantial challenges for effective prevention and therapeutic interventions, consequently imposing severe socioeconomic burdens on human populations and livestock industries.^5,6^ Emerging research has revealed a strong association between intestinal microbial dysbiosis and diarrheal pathogenesis.^7^ Comparative analyses demonstrated marked disparities in microbial community structure, taxonomic abundance, and functional potential between diarrheal patients and healthy counterparts.^8^ Notably, fecal microbiota transplantation from diarrheal donors to germ-free mice has been shown to recapitulate disease phenotypes, including intestinal inflammation and epithelial barrier dysfunction.^9,10^ Nevertheless, the precise mechanisms through which gut microbial dysbiosis in diarrheal patients mediates intestinal inflammatory damage remain to be fully elucidated.

Within the intestinal ecosystem, bidirectional communication between microbiota and the host rarely occurs through direct microbial-cell contact due to the presence of the epithelial barrier.^11^ Extracellular vesicles (EVs), bilayered membrane nanostructures derived from gut microbiota, facilitate intercellular material transport via diverse delivery mechanisms, thereby modulating target cell functionality.^12,13^ Emerging evidence indicated that gut microbial dysbiosis is intricately associated with bovine mastitis development, with microbial EVs serving as crucial functional effectors in this pathogenesis.^14,15^ Furthermore, gut microbiota-derived EVs have been shown to translocate across the intestinal barrier, subsequently exacerbating obesity-associated tissue inflammation and insulin resistance.^16^ Consequently, microbial EVs are recognized as pivotal mediators of both interbacterial and bacteria-host communication. However, whether intestinal barrier dysfunction induced by diarrheal diseases is mediated through gut microbiota-derived EVs remains to be elucidated.

EVs, functioning as critical signaling mediators of gut microbiota, encapsulate bioactive components including RNAs and proteins to mediate host-microbiota crosstalk and inter-tissue communication, exhibiting multifaceted biological capacities in modulating cellular metabolism and inflammatory responses.^17^ miRNAs represent predominant functional constituents within EVs, executing regulatory roles at transcriptional and post-transcriptional levels through formation of miRNA-induced silencing complexes targeting specific genes.^18^ Experimental investigations revealed that miR-200b-3p is upregulated during macrophage polarization, where it binds to the 3‘UTR of p38IP mRNA to suppress its expression, thereby enhancing p38 MAPK activity and promoting macrophage polarization.^19,20^ Clinically, intestinal microbiota-derived let-7b showed significant elevation in inflammatory bowel disease patients, potentiating macrophage-associated proinflammatory cytokine secretion and compromising intestinal epithelial barrier integrity.^21^ Nonetheless, the precise mechanisms underlying how EV-packaged miRNA from diarrheal patients’ gut microbiota disrupt intestinal barrier function, particularly the pathogenically relevant miRNA identities, require systematic investigation.

In the present study, we adopted a microbiota-derived EVs-centric approach to systematically delineate the microbial-mediated mechanisms underlying intestinal inflammatory damage in diarrheal patients. Employing a macrophage-gut epithelial cell interaction axis, we further elucidated the molecular cascade through which EVs-encapsulated miR-125b from diarrheal patients’ gut microbiota orchestrates macrophage polarization, ultimately driving intestinal barrier dysfunction. Our findings provide critical insights into the mechanistic basis of diarrhea-associated intestinal injury, significantly advancing our understanding of gut microbiota-host crosstalk in inflammatory pathologies.

## Materials and methods

### Gut microbiota-derived EVs isolation and identification

Fecal samples were collected from healthy and weaned diarrheal piglets for isolating gut microbiota-derived EVs. The inclusion criteria for weaned piglets (21 days old) in this study involved monitoring post-weaning diarrhea onset to distinguish healthy from diarrheal individuals, based on established protocols from a previous investigation.^22^ Diarrheal piglets were defined as those exhibiting liquid or watery feces for a minimum of two consecutive days, with no prior antibiotic exposure at the time of sample collection. In contrast, healthy piglets showed no history of diarrhea or other illnesses. Using a randomized selection process, we collected fresh fecal samples from 8 diarrheal and 8 healthy piglets. In this study, we first extracted EVs individually from the fecal samples of each donor pig, and then pooled the EVs derived from all healthy or diarrheal donor pigs for subsequent animal and cell treatments. Feces were homogenized in 0.02 μm filtered PBS. EVs were then isolated through differential centrifugation, as described previously.^15^ The process involved centrifuging the homogenate at 340 × g for 10 min at 4°C. The resulting supernatant was subjected to further centrifugation at 10,000 × g for 20 min at 4°C, followed by 18,000 × g for 45 min at 4°C. After passing the mixture through a 0.22 μm filter, a final centrifugation step at 100,000 × g for 2 h at 4°C was performed. The EVs pellet obtained was resuspended in sterile PBS. To confirm the presence of EVs, transmission electron microscopy (TEM) was used. Nanoparticle tracking analysis (NTA) was employed to determine the range of particle diameters. Additionally, Western blotting was conducted to detect the marker proteins of EVs, namely Alix and Calnexin.

### Animals and treatments

Male C57BL/6J mice (6–8 weeks old) were housed under specific pathogen-free (SPF) conditions (22 ± 1°C, 12-h light/dark cycle). Experimental mice were randomly assigned to groups based on body weight, with ad libitum access to food and water. All animal experimental protocols and tissue sampling procedures were approved by the Institutional Animal Care and Use Committee (IACUC) of Huazhong Agricultural University, Hubei Province, China. In this study, all experimental methods strictly adhered to the Guidelines for the Care and Use of Laboratory Animals issued by Huazhong Agricultural University (Ethical Approval Number: HZAUMO-2024–0335).

In the mouse experiment involving oral gavage of EVs, 24 mice were randomly divided into three groups: (i) CON group: received oral gavage with phosphate-buffered saline (PBS); (ii) H-EVs group: received oral gavage with EVs derived from the gut microbiota of healthy pigs; (iii) D-EVs group: received oral gavage with EVs derived from the gut microbiota of diarrheal pigs. The experiment lasted for 7 days, with each mouse administered a daily dose of 0.2 mL. Mice in the H-EVs and D-EVs groups received EVs at a concentration of 50 μg per mouse daily. The dosage of EVs used for treating mice was selected based on our previous research.^9^ At the endpoint, all mice were euthanized, and samples including blood, intestinal segments, and jejunal contents were collected.

To further investigate whether the effects of EVs were associated with the gut microbiota, an additional cohort of 24 mice was subjected to a 14-day antibiotic cocktail regimen in drinking water to deplete the endogenous gut microbiota, followed by repetition of the aforementioned experimental protocol. To induce intestinal microbiota depletion, mice received a broad-spectrum antibiotic cocktail (Meilun Bio., Dalian, China) consisting of streptomycin (1 g/L), ampicillin (0.5 g/L), gentamicin (1 g/L), and vancomycin (0.5 g/L) administered via drinking water. The antibiotics were diluted in drinking water and replenished every other day.^23^ Post-experiment, mice were euthanized, and blood and jejunal segments were harvested for analysis.

In the agomiR mouse experiment, 24 mice were randomly assigned to three groups (*n* = 8): (i) CON group: intraperitoneally injected with 200 μL PBS; (ii) agomiR-NC group: intraperitoneally injected with 200 μL agomiR-NC (2 nmol/10 g body weight); (iii) agomiR-125b group: intraperitoneally injected with 200 μL agomiR-125b (2 nmol/10 g body weight). The nature of agomiR is provided in Table S1. Injections were administered every other day for 5 days. In the macrophage depletion experiment, clodronate liposomes (0.2 mL/mouse, Biohub International Trade Co., Shanghai, China) were administered via intraperitoneal injection to eliminate macrophages. The treatment was initiated 3 days before agomiR-125b administration and repeated every third day thereafter, following a previously established protocol.^24^ At the experimental endpoint, all mice were euthanized, and blood samples and jejunal segments were collected for further analysis.

### Cell culture and treatments

This study utilized murine monocyte-macrophages (RAW264.7) and murine small intestinal epithelial cells (MODE-K). Cells were cultured in DMEM medium (Gibco) supplemented with 10% fetal bovine serum (FBS, Vazyme) and 1% penicillin/streptomycin (Gibco) at 37°C under 5% CO_2_. RAW264.7 or MODE-K cells were seeded 24 hours prior to experiments in 6-well plates or transwell inserts. Post-experiment, supernatants and RAW264.7 cells were collected, and MODE-K cells were harvested using cell scrapers after co-culture.

In the EVs + JSH-23 experiment, three experimental groups (*n* = 3) were established: (i) CON group: RAW264.7 cells received no treatment; (ii) D-EVs group: RAW264.7 cells were treated with diarrheal pig gut microbiota-derived EVs (20 μg/mL); (iii) D-EVs + JSH-23 group: RAW264.7 cells were co-treated with D-EVs (20 μg/mL) and JSH-23 (a NF-κB inhibitor, 10 μM, MedChemexpress).

To elucidate the functional role of miR-125b, RAW264.7 macrophages or MODE-K intestinal epithelial cells were divided into three experimental groups (*n* = 3) and transfected as follows: (i) CON group: untreated control; (ii) Mimics-NC group: transfected with negative control mimics (Mimics-NC, 50 nM, Qingke Biotechnology); (iii) miR-125b-mimics group: transfected with miR-125b mimics (50 nM, Qingke Biotechnology). Transfected cells were maintained for 48 hours under standard culture conditions (37°C, 5% CO_2_). Subsequently, pretreated macrophages were co-cultured with intestinal epithelial cells (MODE-K) for 12 hours in a transwell system to assess intercellular interoperability.

In the D-EVs and inhibitors experiments, macrophages were divided into the following three groups (*n* = 3): (i) CON group: untreated; (ii) D-EVs group: treated with extracellular vesicles derived from diarrheal pig gut microbiota (D-EVs, 20 μg/mL); (iii) D-EVs + miR-125b inhibitors group: co-treated with D-EVs (20 μg/mL) and miR-125b inhibitors (100 nM, Qingke Biotechnology). Following a 48-hour incubation period, cell culture supernatants and RAW264.7 cells were harvested for subsequent analysis. To investigate the impact of D-EVs and inhibitors-treated macrophages on intestinal epithelial cells, RAW264.7 cells treated with either D-EVs alone or D-EVs combined with inhibitors were placed in the basolateral compartment of 6-well plates, while MODE-K cells were seeded in the apical compartment of transwell inserts. Following 12 hours of co-culture under standardized conditions (37°C, 5% CO_2_), MODE-K cells were collected. The nature of miR-125b-mimics and inhibitors is provided in Table S1.

### Primary macrophage culture and macrophage adoptive transfer

Primary peritoneal macrophages were isolated from mice according to the protocol described by previous study,^25^ with minor modifications. Mice were sacrificed and sterilized externally by immersion in 75% ethanol. The skin of the abdominal region was carefully incised to expose the intact peritoneal wall. A total of 5 mL of complete DMEM medium (without antibiotics) was gently injected into the peritoneal cavity using a sterile 5-mL syringe. The mice were then gently massaged for approximately 5 minutes to facilitate the detachment of peritoneal cells. After incubation, the peritoneal fluid was carefully aspirated using syringe and collected into a sterile 15-mL centrifuge tube. The suspension was centrifuged at 1000 rpm for 5 minutes at room temperature, and the supernatant was discarded. The cell pellet was resuspended in phosphate-buffered saline (PBS), centrifuged again under the same conditions, and washed twice to remove debris. Following the final wash, cells were resuspended in complete medium and seeded into tissue culture plates. After a 4-hour incubation at 37°C in a humidified 5% CO₂ incubator, non-adherent cells were removed by gently replacing the medium with fresh antibiotic-free complete medium. The remaining adherent cells, primarily peritoneal macrophages, were maintained overnight and subjected to transfection the following day. In the transfection experiment using primary macrophages, the grouping and treatment protocols were consistent with those applied to miR-125b-transfected RAW264.7 cells. Supernatants and primary macrophages were collected 48 hours post-transfection.

For the adoptive transfer experiment, 24 mice were randomly allocated to three groups (*n* = 8): (i) CON group: intraperitoneally injected with 200 μL PBS; (ii) Untreated group: intraperitoneally administered untreated primary macrophages (1 × 10^5^ cells per mouse); (iii) Treated group: intraperitoneally administered primary macrophages pretreated with miR-125b mimics (1 × 10^5^ cells per mouse). At 24 hours post-injection, all mice were euthanized, and blood samples along with jejunal segments were harvested for downstream analysis.

### Cellular uptake of EVs

RAW 264.7 macrophages were equilibrated in serum-free Hank’s balanced salt solution (HBSS, Gibco, #14175095) for 15 min at 37°C under 5% CO₂ to synchronize cellular metabolic states. Lipophilic dye DiI (Sigma-Aldrich, #42364)-labeled gut microbiota-derived EVs were introduced, followed by 12-h co-incubation in Opti-MEM reduced-serum medium (Gibco, #31985070) to minimize nonspecific binding. Post-incubation, cells were washed thrice with ice-cold PBS containing 0.1% Tween-20 to remove uninternalized EVs. A fluorescent microscope was used to analyze the DiI-positive cells.

### Histological analyses

The H&E staining assay was conducted following the methodology outlined in prior research.^10,26^ Briefly, jejunal tissue samples were fixed in 4% paraformaldehyde for 24 h at 4°C, paraffin-embedded, and sectioned at 4 μm thickness for hematoxylin and eosin (H&E) staining using a standardized protocol optimized for mucosal architecture preservation. Morphometric analysis was conducted in a blinded manner using CaseViewer software (version 220 2.2) with calibrated measurement tools. Villus height was defined as the distance from the villus tip to the crypt-villus junction, while crypt depth extended from the junction to the basal lamina. To ensure accuracy and objectivity, we randomly selected five sections from each animal’s jejunal samples to measure villus height and crypt depth separately, with the final values representing the mean of these measurements.

### Enzyme-linked immunosorbent assay (ELISA)

Serum, jejunal homogenates (prepared in RIPA buffer with protease inhibitors, centrifuged at 12,000×g for 15 min at 4°C), and cell culture supernatants (filtered through 0.22 μm PVDF membranes) were analyzed for biomarkers of intestinal barrier dysfunction (diamine oxidase (DAO), d-lactic acid (D-LA)) and inflammatory mediators (interleukin (IL)-1β, IL-6, IL-10, tumor necrosis factor (TNF)-α) using species-specific ELISA kits in accordance with the manufacturer’s instructions (Shanghai Enzyme-linked Biotechnology Co. Ltd., Shanghai, China). All assays were performed in technical triplicates with freshly thawed aliquots to avoid freeze-thaw artifacts.

### Quantification of RNA

Jejunal tissue samples were homogenized in TRIzol™ Reagent using a Precellys Evolution tissue disruptor with ceramic beads. RNA was purified via chloroform-phase separation followed by isopropanol precipitation, with on-column DNase I digestion to eliminate genomic DNA contamination. RNA integrity was rigorously assessed using an Agilent 2100 Bioanalyzer with RNA Nano Chips, while concentration and purity were quantified via NanoDrop OneC. For cDNA synthesis, 1 μg of total RNA underwent reverse transcription using the StarScript III All-in-one RT Mix. Quantitative PCR was performed on a LightCycler 480 II. Expression normalization employed β-actin as the reference gene, relative quantification used the 2^−ΔΔCt^ method. Primer information is provided in Table S2.

### Western blot analyses

Jejunal tissue specimens were homogenized in ice-cold RIPA lysis buffer supplemented with 1× protease/phosphatase inhibitor cocktail, followed by centrifugation at 15,000 × g for 20 min at 4°C to collect soluble protein fractions. Protein concentrations were determined via micro-BCA assay using bovine serum albumin standards with absorbance measured at 562 nm on a Multiskan Sky microplate reader. For immunoblotting, denatured proteins were resolved on SDS-PAGE gels and proteins were electrophoretically transferred to nitrocellulose membranes. Membranes were blocked for 2 h at room temperature, then incubated overnight at 4°C with primary antibodies. After three 10-min TBST washes, membranes were probed with HRP-conjugated secondary antibodies for 2 h at room temperature. Finally, protein expression was measured and evaluated using an Imaging System (Bio-Rad, USA) and Quantity One software (Bio-Rad, USA). Table S3 contains information about the antibodies.

### 16S rRNA sequencing

Fecal/jejunal digesta-derived genomic DNA was isolated using the QIAamp Fast DNA Stool Mini Kit (Qiagen, Germany), followed by amplification and sequencing of the hypervariable V3-V4 regions of the 16S rRNA gene on an Illumina MiSeq system, which generated paired-end sequences.^27^ Raw sequencing data were demultiplexed based on sample-specific barcodes, followed by quality filtering to retain high-fidelity sequences using Trimmomatic (v0.39) with sliding-window trimming (4-bp window, average Phred score > 20). High-quality sequences were processed using the QIIME2 platform (v2022.2), implementing the DADA2 algorithm for error correction and amplicon sequence variant (ASV) generation.^28^ Taxonomic classification was performed via a Naïve Bayes classifier trained on the SILVA132 reference database (99% identity threshold) with a minimum confidence threshold of 0.8 for taxonomic assignment.

### miRNA sequencing and target analysis

EVs derived from gut microbiota were isolated using the RNA pure extraction kit (Aidlid, Beijing, China). The miRNA library was prepared with the Small RNA Prep Kit (Illumina, California, USA) for miRNA sequencing. RNA sequencing was performed by Shanghai Majorbio company, China, to obtain the miRNA profiles in EVs. Sequencing data were analyzed via the Majorbio Cloud Platform (www.majorbio.com). To predict the target genes of miRNAs, the 3’-UTRs of mRNAs were scanned using Hybrid (bibiserv.cebitec.uni-bielefeld.de/rnahybrid), which predicts miRNA-mRNA complementary pairing.

### Statistical analysis

The data are reported as means ± SEM. For comparisons among more than two groups, a one-way analysis of variance (ANOVA) and a post hoc Tukey test were performed (SPSS version 20.0 for Windows; SPSS Inc., Chicago, IL, USA). Differences for which *p* < 0.05 were considered to be statistically significant. The numbers of replicates used to perform statistical analyses are noted in the relevant figure legends.

## Results

### Isolation and characterization of gut microbiota-derived EVs from healthy and diarrheal piglets

**Figure 1.**
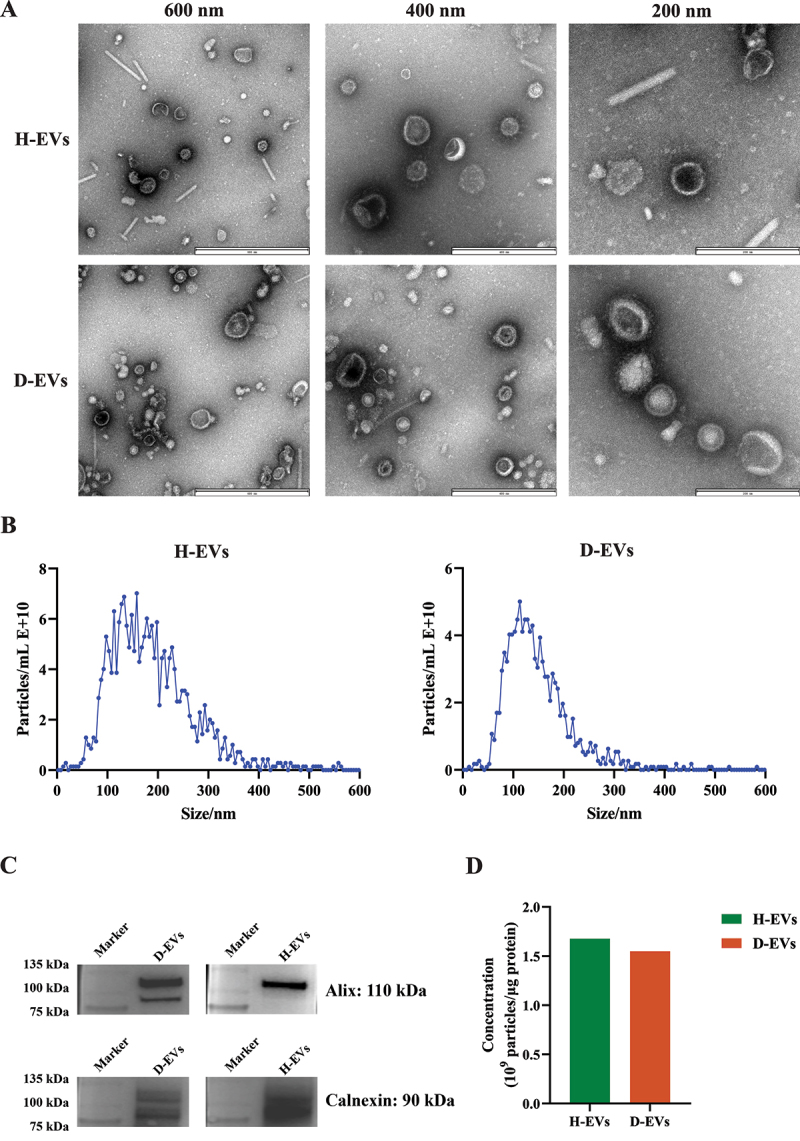
Isolation and characterization of gut microbiota-derived EVs from healthy and diarrheal piglets. (A) Representative TEM images of H-EVs and D-EVs showing intact cup-shaped structures with bilayer membranes (scale bar: 600, 400, and 100 nm). (B) NTA of EVs size distribution. (C) Western blot analysis of EVs-specific markers (Alix and Calnexin) in H-EVs and D-EVs. (D) quantification of EVs particle concentration (particles/μg protein).

To unravel the mystery of how gut microbiota-derived EVs influence host intestinal health, we first isolated EVs from the intestinal microbiota of healthy and diarrheal piglets and performed biological characterization. As shown in Figure 1A, both healthy (H-EVs) and diarrheal (D-EVs) piglet-derived EVs exhibited typical intact cup-shaped membrane vesicles with bilayer structures and sizes revealed by transmission electron microscopy (TEM). Nanoparticle tracking analysis (NTA) demonstrated that the average diameters of H-EVs and D-EVs were 189.6 nm and 148.2 nm, respectively (Figure 1B). To further characterize the gut microbiota-derived EVs we prepared, western blotting confirmed the expression of EV-specific markers Alix and Calnexin in both H-EVs and D-EVs (Figure 1C). As shown in Figure 1D, no significant difference in particle concentration was observed between H-EVs and D-EVs (1.68 × 10^9^ particles/μg vs 1.55 × 10^9^ particles/μg protein). These results indicate that gut microbiota-derived EVs from healthy and diarrheal piglets were successfully obtained, with no quantitative differences between the two groups.

### Diarrhea-associated microbial EVs trigger pro-inflammatory macrophage polarization and intestinal barrier injury in SPF mice

**Figure 2.**
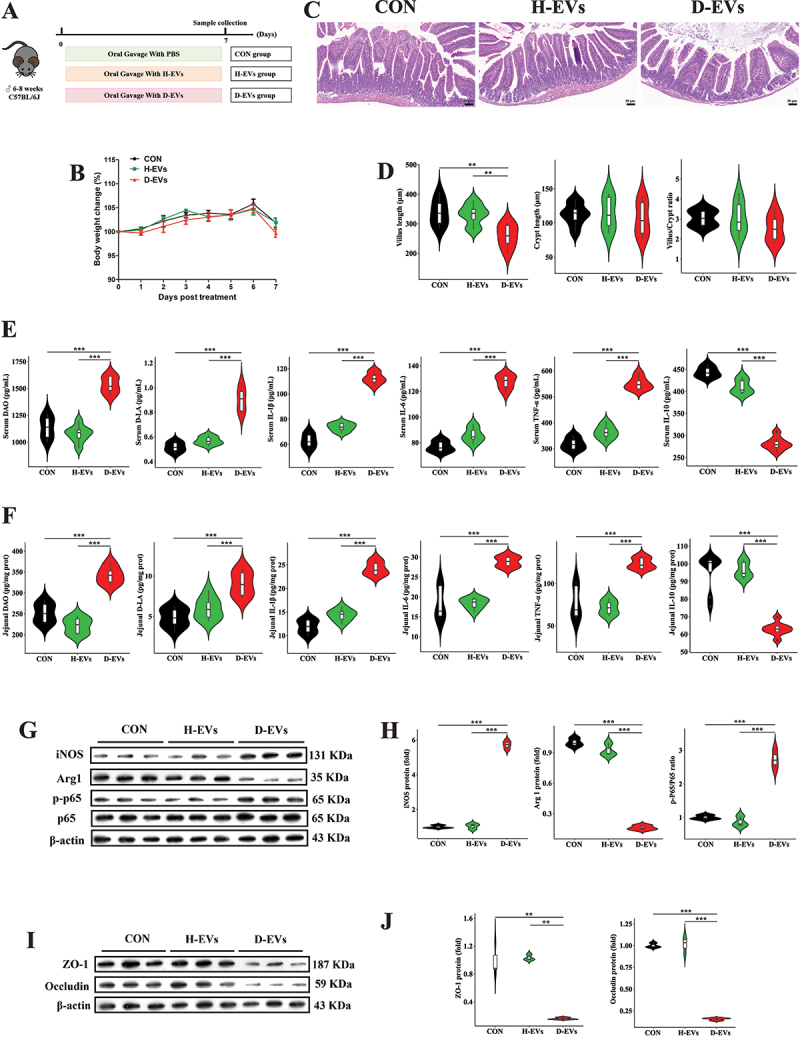
D-EVs induce pro-inflammatory responses and intestinal barrier dysfunction in SPF mice. (A) experimental design of oral EVs administration. (B) longitudinal monitoring of animal body mass variations. (C,D) histopathological evaluation (scale bar, 50 μm) and morphometric analysis of jejunal epithelium using hematoxylin-eosin staining. (E,F) quantitative determination of intestinal permeability biomarkers (diamine oxidase, D-lactate) and pro-/anti-inflammatory cytokines (IL-1β, IL-6, TNF-α, IL-10) in systemic circulation and local tissue. (G,H) immunoblotting detection of M1/M2 macrophage polarization markers (inducible nitric oxide synthase, arginase-1) and NF-κB signaling activation (phosphorylated p65 subunit). (I,J) tight junction protein expression profiling (zona occludens-1, occludin) in intestinal epithelium. Experimental groups comprised 8 biological replicates for physiological and cytokine measurements, and 3 technical replicates for immunoblot analyses. Statistical differences were assessed using one-way ANOVA with Tukey’s post hoc comparison, with data presented as mean values ± standard error of the mean. Significance thresholds: **p < 0.01, ***p < 0.001.

To investigate the effects of gut microbiota-derived EVs on intestinal inflammation and barrier function, 24 mice were divided into three groups and orally gavaged daily with equal amounts of H-EVs, D-EVs, or PBS for 7 days (Figure 2A). Although no significant differences in body weight changes were observed among the three groups throughout the experimental period (Figure 2B), D-EVs markedly decreased jejunal villus length in mice at the end of the experiment (Figure 2C and D). Additionally, serum and jejunal tissue concentrations of DAO, D-LA, IL-1β, IL-6, and TNF-α in the D-EVs group were significantly higher than those in the control and H-EVs groups, while IL-10 levels were significantly lower (Figure 2E and F). To further examine whether D-EVs caused intestinal inflammatory injury, we detected intestinal macrophage polarization markers and tight junction protein expression. D-EVs treatment significantly increased protein expression of inducible nitric oxide synthase (iNOS) and p-NF-κB p65 in the jejunum while decreasing arginase-1 (Arg1) expression (Figure 2G and H). Furthermore, the protein expression of tight junction proteins zonula occludens-1 (ZO-1) and Occludin was also significantly reduced in the D-EVs group (Figure 2I and J). We further analyzed the fecal and jejunal microbiota of the three groups of mice. The α-diversity of the fecal and jejunal microbiota were similar across all groups (Figure S1A, Figure S2A). There was no difference in the β-diversity of fecal microbiota among the three groups (Figure S1B), while the β-diversity of the jejunal microbiota was significantly different among the three groups (Figure S2B). The genus-level taxonomic composition of the fecal and jejunal microbiota is shown in Figure S1C and Figure S2C. Moreover, only a few fecal and jejunal bacterial genera exhibited differences in abundance at the genus level among the groups (Figure S1D-E, Figure S2D-E). These results demonstrate that D-EVs contribute to intestinal inflammatory injury in mice.

### Diarrhea-associated microbial EVs trigger pro-inflammatory macrophage polarization and intestinal barrier injury in pseudo-germ-free mice

**Figure 3.**
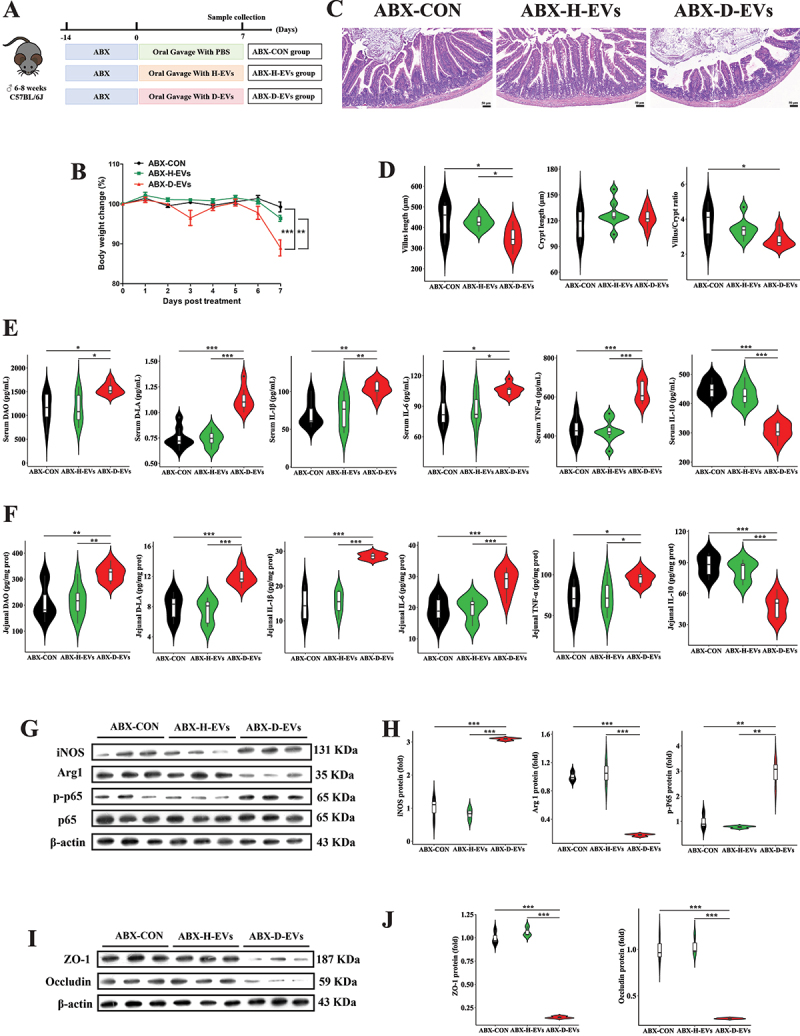
D-EVs induce pro-inflammatory responses and intestinal barrier dysfunction in pseudo-germ-free mice. (A) experimental design of oral EVs administration. (B) longitudinal monitoring of animal body mass variations. (C,D) histopathological evaluation (scale bar, 50 μm) and morphometric analysis of jejunal epithelium using hematoxylin-eosin staining. (E,F) quantitative determination of intestinal permeability biomarkers (diamine oxidase, D-lactate) and pro-/anti-inflammatory cytokines (IL-1β, IL-6, TNF-α, IL-10) in systemic circulation and local tissue. (G,H) immunoblotting detection of M1/M2 macrophage polarization markers (inducible nitric oxide synthase, arginase-1) and NF-κB signaling activation (phosphorylated p65 subunit). (I,J) tight junction protein expression profiling (zona occludens-1, occludin) in intestinal epithelium. Experimental groups comprised 8 biological replicates for physiological and cytokine measurements, and 3 technical replicates for immunoblot analyses. Statistical differences were assessed using one-way ANOVA with Tukey’s post hoc comparison, with data presented as mean values ± standard error of the mean. Significance thresholds: **p < 0.05, ***p < 0.01, *p < 0.001.

To determine whether gut microbiota participates in D-EVs-mediated intestinal inflammatory injury, 24 mice pretreated with antibiotics to eliminate gut microbiota were divided into three groups and orally gavaged daily with equal amounts of H-EVs, D-EVs, or PBS for 7 days (Figure 3A). Interestingly, body weight changes in the ABX-D-EVs group were significantly lower than those in the other two groups (Figure 3B). D-EVs treatment substantially lowered jejunal villus length and villus/crypt ratio in mice (Figure 3C and D). Additionally, serum and jejunal tissue concentrations of DAO, D-LA, IL-1β, IL-6, and TNF-α in the ABX-D-EVs group were significantly higher than those in the control and H-EVs groups, while IL-10 levels were significantly lower (Figure 3E and F). To further investigate whether D-EVs caused intestinal inflammatory injury in pseudo-germ-free mice, we detected intestinal macrophage polarization markers and tight junction protein expression. D-EVs treatment significantly increased protein expression of iNOS and p-NF-κB p65 in the jejunum while decreasing Arg1 expression (Figure 3G and H). Simultaneously, the protein expression of tight junction proteins ZO-1 and Occludin was also markedly decreased in the ABX-D-EVs group (Figure 3I and J). These results indicate that D-EVs-induced intestinal inflammatory injury in mice does not require gut microbiota involvement.

### NF-κB activation mediates diarrhea-associated microbial EVs induced pro-inflammatory macrophage polarization

**Figure 4.**
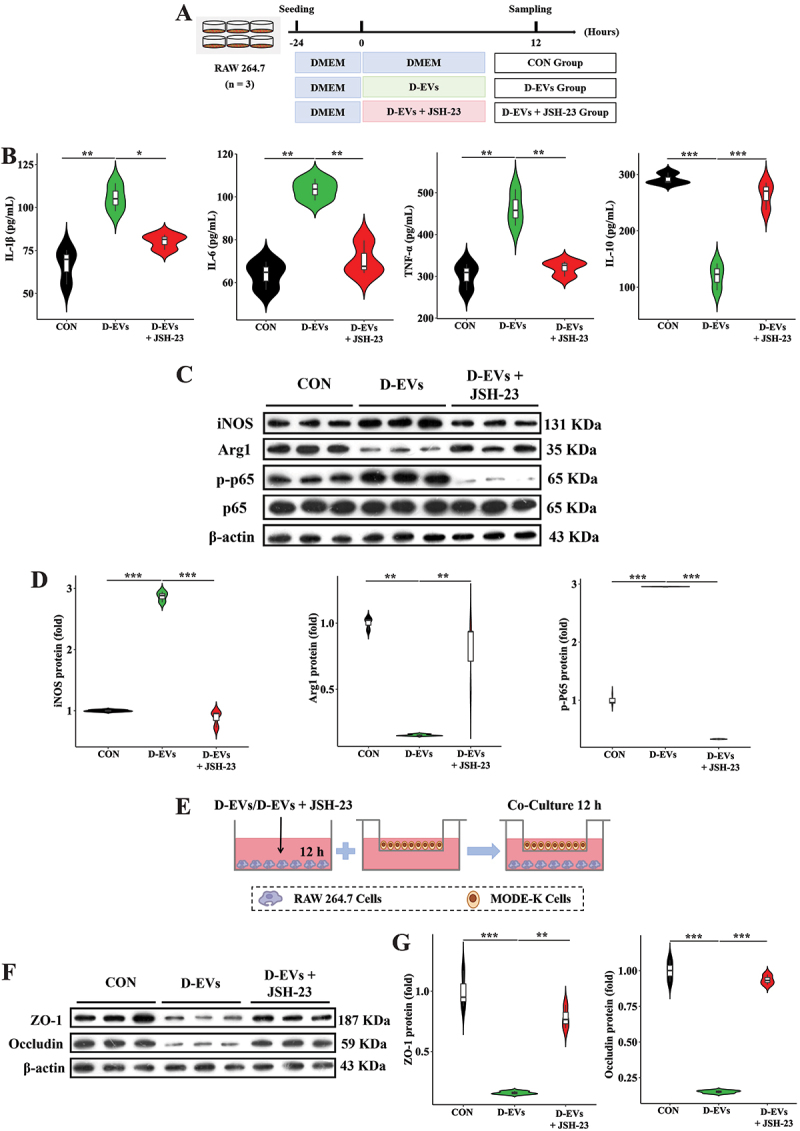
NF-κB activation is required for D-EV-induced macrophage polarization. (A) experimental workflow of JSH-23 (NF-κB inhibitor) treatment. (B) secretory profiles of pro-/anti-inflammatory mediators (IL-1β, IL-6, TNF-α, IL-10) quantified in macrophage-conditioned culture media. (C,D) immunoblotting characterization of M1/M2 phenotypic markers (inducible nitric oxide synthase vs. arginase-1) and nuclear factor-kappa B activation state (phosphorylated p65 subunit quantification). (E) schematic representation of transwell co-culture model establishing macrophage-epithelial paracrine signaling. (F,G) functional evaluation of epithelial barrier integrity through tight junction regulators (zona occludens-1 and occludin) immunodetection. Experimental validation included triplicate experimental sets with quantitative data normalized to reference controls. Statistical differences were assessed using one-way ANOVA with Tukey’s post hoc comparison, with data presented as mean values ± standard error of the mean. Significance thresholds: **p < 0.05, ***p < 0.01, *p < 0.001.

We further explored how gut microbiota-derived EVs cause intestinal inflammatory injury *in vitro*. Macrophage uptake experiments showed that EVs could be directly internalized into the cytoplasm of macrophages (Figure S3). We utilized the NF-κB p65-specific inhibitor JSH-23 to investigate how D-EVs induce pro-inflammatory macrophage polarization (Figure 4A). D-EVs treatment significantly increased concentrations of IL-1β, IL-6, and TNF-α while decreasing IL-10 in macrophage supernatants, and these effects were reversed in the D-EVs + JSH-23 group (Figure 4B). Additionally, D-EVs treatment significantly upregulated iNOS and p-NF-κB p65 protein expression while downregulating Arg1 in macrophages, which was also reversed by JSH-23 (Figure 4C and D). To further study macrophage-epithelial cell interactions, we conducted a co-culture experiment (Figure 4E). In the co-culture system, protein expression of ZO-1 and Occludin in intestinal epithelial cells was decreased in the D-EVs group but restored in the presence of JSH-23 (Figure 4F and G). These results demonstrate that NF-κB activation is a central event in D-EVs-induced pro-inflammatory macrophage polarization.

### Comparative miRNA profiling identifies miR-125b as a key regulator of pro-inflammatory polarization

**Figure 5.**
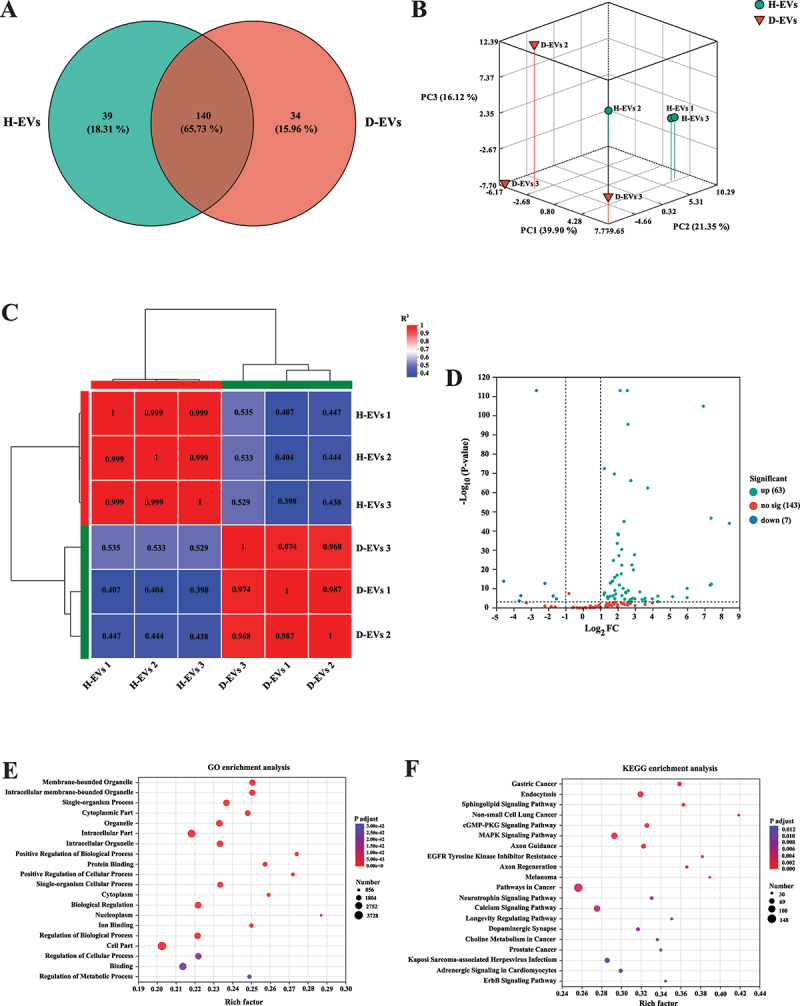
Comparative miRNA profiling of H-EVs and D-EVs. (A) venn diagram of shared and unique miRnas. (B) Pearson correlation heatmap of miRNA profiles. (C) PCA plot of miRNA profiles. (D) volcano plot of differentially expressed miRnas. (E) GO pathway enrichment analysis. (F) KEGG pathway enrichment analysis. *n* = 3.

To explore whether miRNA components in diarrheal donor gut microbiota-derived EVs participate in promoting pro-inflammatory macrophage polarization, we performed miRNA sequencing and analysis. As shown in Figure 5A, 140 miRNAs were co-expressed in H-EVs and D-EVs, while 39 and 34 miRNAs were unique to H-EVs and D-EVs, respectively. Pearson correlation and PCA analyses revealed substantial differences in miRNA composition between H-EVs and D-EVs (Figure 5B and C). Compared to H-EVs, 63 miRNAs were upregulated and 7 miRNAs were downregulated in D-EVs (Figure 5D and Table S4). GO and KEGG enrichment analyses of these differentially expressed miRNAs are shown in Figure 5E and F.

**Figure 6.**
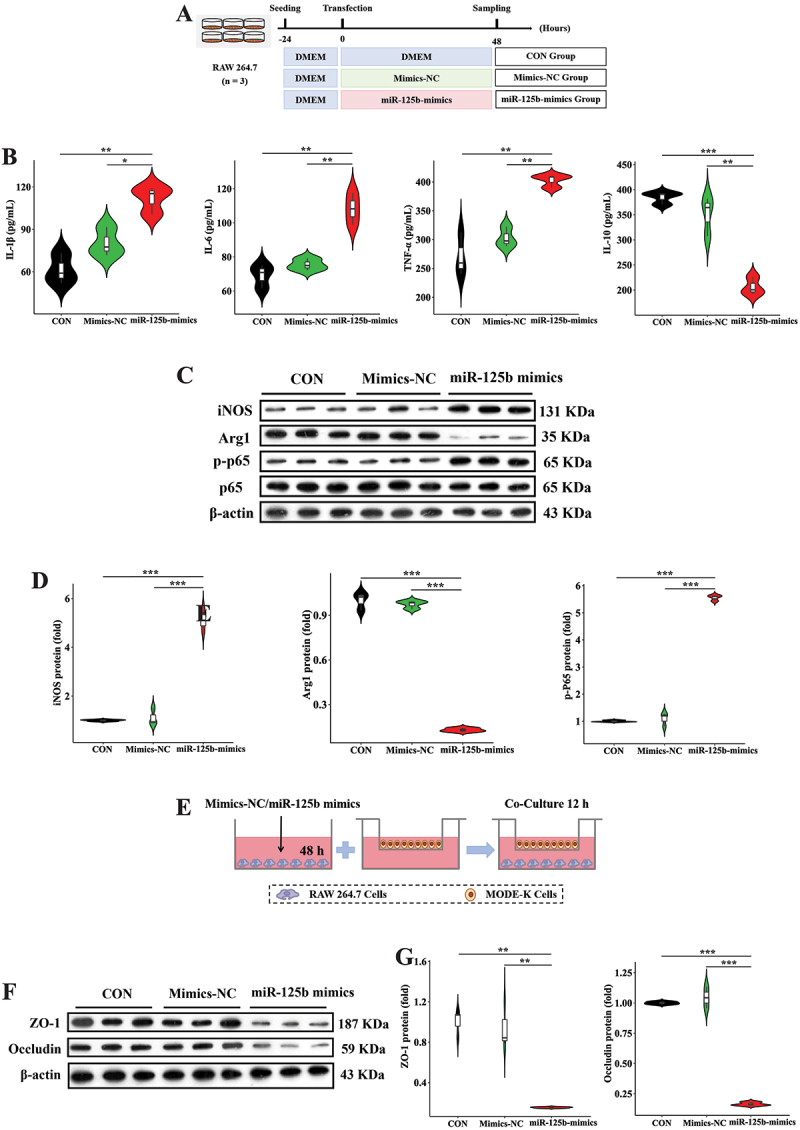
miR-125b induced pro-inflammatory effects. (A) experimental workflow of miR-125b treatment. (B) secretory profiles of pro-/anti-inflammatory mediators (IL-1β, IL-6, TNF-α, IL-10) quantified in macrophage-conditioned culture media. (C,D) immunoblotting characterization of M1/M2 phenotypic markers (inducible nitric oxide synthase vs. arginase-1) and nuclear factor-kappa B activation state (phosphorylated p65 subunit quantification). (E) schematic representation of transwell co-culture model establishing macrophage-epithelial paracrine signaling. (F,G) functional evaluation of epithelial barrier integrity through tight junction regulators (zona occludens-1 and occludin) immunodetection. Experimental validation included triplicate experimental sets with quantitative data normalized to reference controls. Statistical differences were assessed using one-way ANOVA with Tukey’s post hoc comparison, with data presented as mean values ± standard error of the mean. Significance thresholds: **p < 0.05, ***p < 0.01, *p < 0.001.

**Figure 7.**
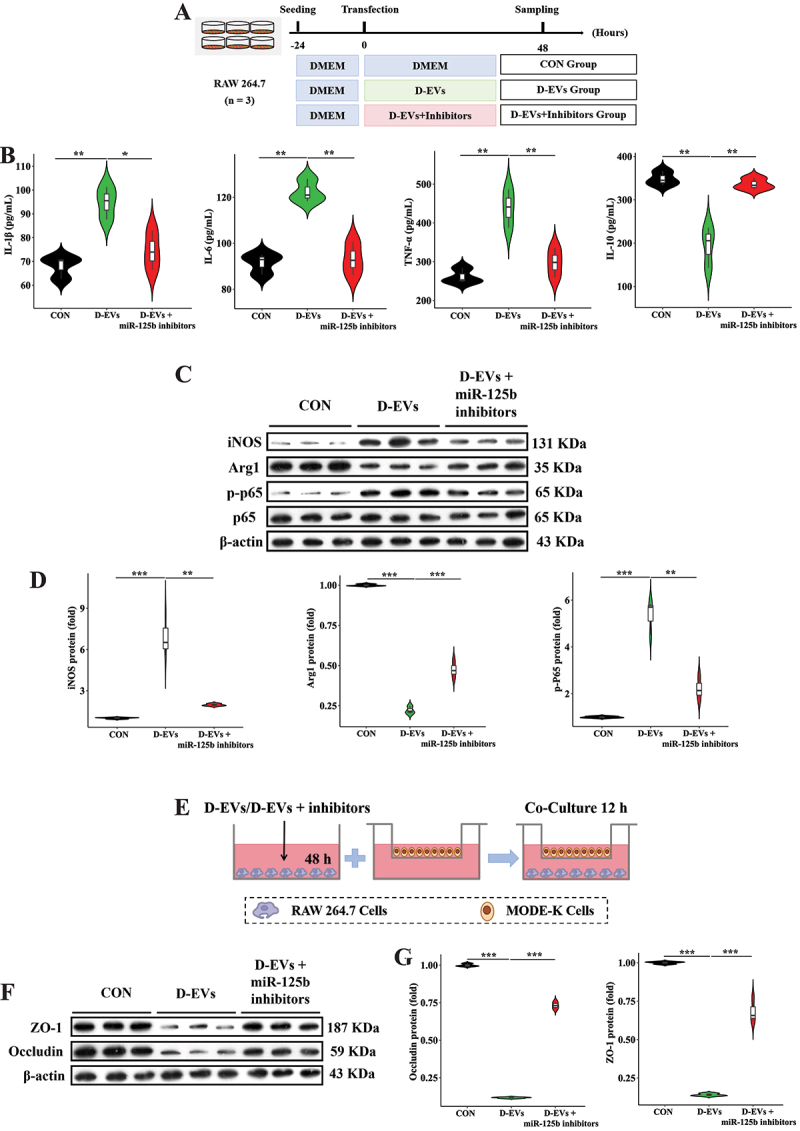
miR-125b inhibitors reverse D-EVs-induced phenotypes. (A) experimental workflow of miR-125b inhibitors treatment. (B) secretory profiles of pro-/anti-inflammatory mediators (IL-1β, IL-6, TNF-α, IL-10) quantified in macrophage-conditioned culture media. (C,D) immunoblotting characterization of M1/M2 phenotypic markers (inducible nitric oxide synthase vs. arginase-1) and nuclear factor-kappa B activation state (phosphorylated p65 subunit quantification). (E) schematic representation of transwell co-culture model establishing macrophage-epithelial paracrine signaling. (F,G) functional evaluation of epithelial barrier integrity through tight junction regulators (zona occludens-1 and occludin) immunodetection. Experimental validation included triplicate experimental sets with quantitative data normalized to reference controls. Statistical differences were assessed using one-way ANOVA with Tukey’s post hoc comparison, with data presented as mean values ± standard error of the mean. Significance thresholds: **p < 0.05, ***p < 0.01, *p < 0.001.

To investigate the impact of specific miRNAs in D-EVs on pro-inflammatory macrophage polarization, we selected miR-125b, which was significantly enriched in D-EVs, for *in vitro* experiments (Figure 6A). miR-125b treatment significantly increased IL-1β, IL-6, and TNF-α concentrations while decreasing IL-10 levels in macrophage supernatants (Figure 6B). Additionally, miR-125b upregulated iNOS and p-NF-κB p65 protein expression while downregulating Arg1 in macrophages (Figure 6C and D). Further study showed that protein expression of ZO-1 and Occludin in intestinal epithelial cells was substantially lowered in the miR-125b group in the co-culture system (Figure 6F and G). We further employed miR-125b inhibitors to validate the role of miRNAs in pro-inflammatory macrophage polarization (Figure 7A). miR-125b inhibitors significantly reversed D-EVs-induced changes in IL-1β, IL-6, TNF-α, and IL-10 concentrations in macrophage supernatants (Figure 7B). Moreover, miR-125b inhibitors restored the protein expression of iNOS, p-NF-κB p65, and Arg1 in D-EVs-treated macrophages (Figure 7C and D). Furthermore, protein expression of ZO-1 and Occludin in intestinal epithelial cells was markedly reversed with miR-125b inhibitors in the co-culture system (Figure 7F and G). We further treated MODE-K cells with miR-125b to reveal its direct impact on intestinal epithelial cells (Figure S4A). Compared with the CON and NC groups, miR-125b did not significantly alter the gene expression of IL-1β, IL-6, TNF-α, and IL-10 in intestinal epithelial cells (Figure S4B). In addition, there were no significant differences in the protein expression of ZO-1 and Occludin in intestinal epithelial cells among the three groups (Figure S4C and D). These results indicate that miR-125b participates in D-EVs-mediated regulation of pro-inflammatory macrophage polarization.

### miR-125b disrupts the intestinal homeostasis by activating NF-κB via targeting NF-κBIA to drive pro-inflammatory macrophage polarization

**Figure 8.**
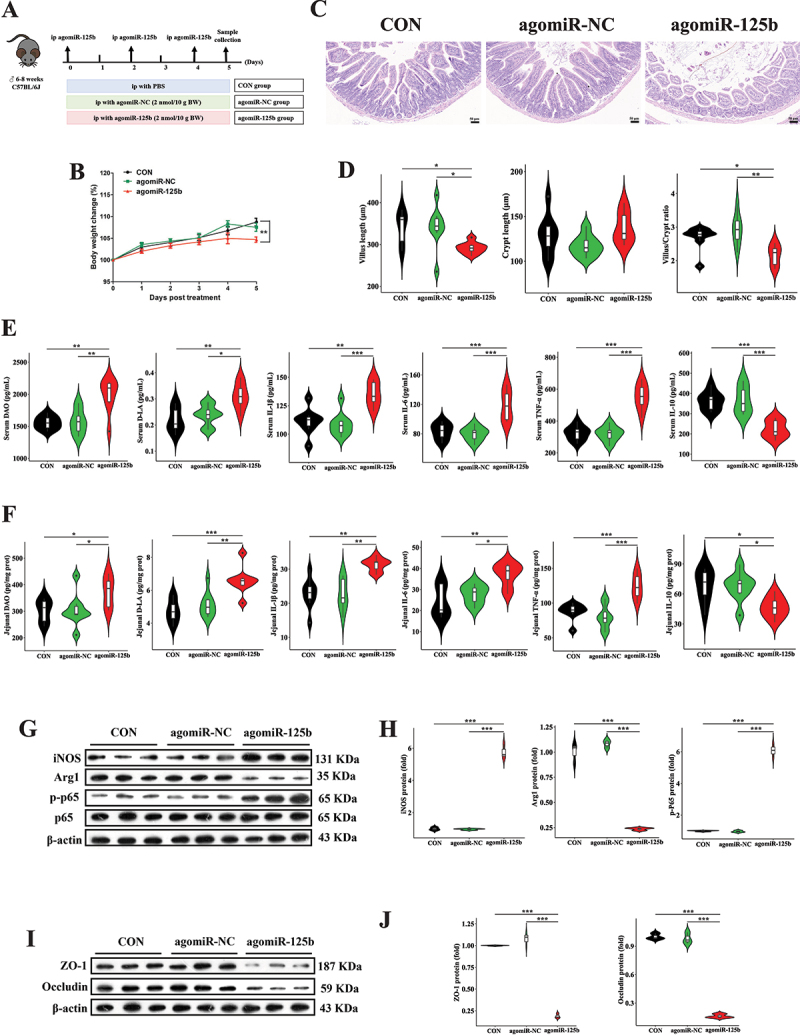
Overexpression of miR-125b induce pro-inflammatory responses and intestinal barrier dysfunction. (A) AgomiR-125b administration protocol. (B) longitudinal monitoring of animal body mass variations. (C,D) histopathological evaluation (scale bar, 50 μm) and morphometric analysis of jejunal epithelium using hematoxylin-eosin staining. (E,F) quantitative determination of intestinal permeability biomarkers (diamine oxidase, D-lactate) and pro-/anti-inflammatory cytokines (IL-1β, IL-6, TNF-α, IL-10) in systemic circulation and local tissue. (G,H) immunoblotting detection of M1/M2 macrophage polarization markers (inducible nitric oxide synthase, arginase-1) and NF-κB signaling activation (phosphorylated p65 subunit). (I,J) tight junction protein expression profiling (zona occludens-1, occludin) in intestinal epithelium. Experimental groups comprised 8 biological replicates for physiological and cytokine measurements, and 3 technical replicates for immunoblot analyses. Statistical differences were assessed using one-way ANOVA with Tukey’s post hoc comparison, with data presented as mean values ± standard error of the mean. Significance thresholds: **p < 0.05, ***p < 0.01, *p < 0.001.

To investigate the mechanism by which miR-125b promotes pro-inflammatory macrophage polarization and intestinal epithelial barrier disruption, we performed intraperitoneal injection of agomiR-125b in mice (Figure 8A). Body weight changes in the agomiR-125b group were significantly lower than those in the other groups (Figure 8B). AgomiR-125b treatment reduced jejunal villus length and villus/crypt ratio (Figure 8C and D). Additionally, serum and jejunal tissue concentrations of DAO, D-LA, IL-1β, IL-6, and TNF-α were significantly elevated in the agomiR-125b group, while IL-10 levels were reduced (Figure 8E and F). Concurrently, agomiR-125b treatment significantly increased protein expression of iNOS and p-NF-κB p65 while decreasing Arg1 expression in the jejunum (Figure 8G and H). Furthermore, protein expression of tight junction proteins ZO-1 and Occludin was also substantially lowered in the agomiR-125b group (Figure 8I and J). We further employed a macrophage depletion mouse model to explore the central role of macrophages in the miR-125b induced disruption of intestinal homeostasis (Figure S5A). There were no significant differences in body weight changes among the three groups of mice (Figure S5B). AgomiR-125b treatment did not cause changes in the morphology of the jejunum in macrophage-depleted mice (Figure S5C and D). In addition, there were no significant differences in the concentrations of DAO, D-LA, IL-1β, IL-6, and TNF-α in the serum and jejunum tissues among the three groups of mice (Figure S5E and F). Moreover, the protein expression of ZO-1 and Occludin in the jejunum of mice in the agomiR-125b group was not different from that of the other two groups (Figure S5G and H). Prediction analysis using the Hybrid platform suggested that NF-κBIA, a negative regulator of NF-κB signaling, might be a target gene of miR-125b. A potential binding site for miR-125b was identified at position 226 of the NF-κBIA 3’ UTR (Figure S6A). Subsequent gene expression analysis confirmed significant downregulation of NF-κBIA in the jejunum of the agomiR-125b group (Figure S6B). These results demonstrate that NF-κBIA is involved in miR-125b-induced intestinal inflammatory injury.

### Adoptive transfer of miR-125b-primed macrophages exacerbates intestinal injury

**Figure 9.**
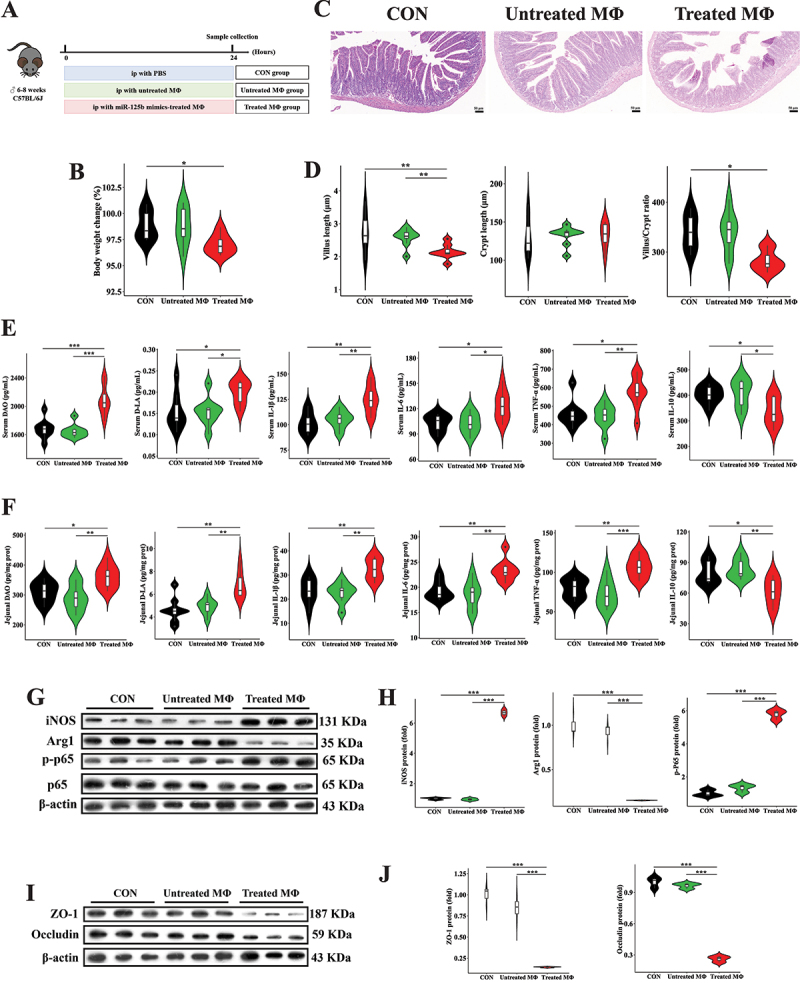
Adoptive transfer of miR-125b-primed macrophages induces intestinal injury. (A) experimental design illustrating macrophage adoptive transfer protocol. (B) longitudinal monitoring of animal body mass variations. (C,D) histopathological evaluation (scale bar, 50 μm) and morphometric analysis of jejunal epithelium using hematoxylin-eosin staining. (E,F) quantitative determination of intestinal permeability biomarkers (diamine oxidase, D-lactate) and pro-/anti-inflammatory cytokines (IL-1β, IL-6, TNF-α, IL-10) in systemic circulation and local tissue. (G,H) immunoblotting detection of M1/M2 macrophage polarization markers (inducible nitric oxide synthase, arginase-1) and NF-κB signaling activation (phosphorylated p65 subunit). (I,J) tight junction protein expression profiling (zona occludens-1, occludin) in intestinal epithelium. Experimental groups comprised 8 biological replicates for physiological and cytokine measurements, and 3 technical replicates for immunoblot analyses. Statistical differences were assessed using one-way ANOVA with Tukey’s post hoc comparison, with data presented as mean values ± standard error of the mean. Significance thresholds: **p < 0.05, ***p < 0.01, *p < 0.001.

To further validate the role of miR-125b in promoting pro-inflammatory macrophage polarization, we isolated primary peritoneal macrophages from mice and treated them with miR-125b (Figure S7A). Consistently, miR-125b treatment significantly increased IL-1β, IL-6, and TNF-α concentrations while decreasing IL-10 levels in primary macrophage supernatants (Figure S7B). Additionally, miR-125b upregulated iNOS and p-NF-κB p65 protein expression while downregulating Arg1 in primary macrophages (Figure S7C and D). We further conducted adoptive transfer experiments by administering miR-125b-treated primary macrophages to recipient mice (Figure 9A). As shown in Figure S8, NF-κBIA gene expression in the jejunum of the adoptive transfer group was significantly lower than in the other groups. Body weight changes in the adoptive transfer of miR-125b-treated primary macrophages group were significantly lower than those in the CON groups (Figure 9B). Adoptive transfer of miR-125b-treated primary macrophages significantly reduced jejunal villus length at the end of the experiment (Figure 9C and D). Additionally, serum and jejunal tissue concentrations of DAO, D-LA, IL-1β, IL-6, and TNF-α were significantly elevated in the adoptive transfer group, while IL-10 levels were reduced (Figure 9E and Figure F). Concurrently, protein expression of iNOS and p-NF-κB p65 was significantly increased, and Arg1 expression was decreased in the jejunum of mice receiving miR-125b-treated macrophages (Figure 9G and H). Furthermore, protein expression of tight junction proteins ZO-1 and Occludin was also markedly decreased in the adoptive transfer group (Figure 9I and J). These results reaffirm the mechanism by which miR-125b induces intestinal inflammatory injury.

## Discussion

Diarrheal diseases afflict millions of patients globally, incurring annual healthcare expenditure losses amounting to billions of US dollars.^29^ While mechanistic investigations spanning decades have significantly reduced diarrheal mortality, persistent intestinal inflammation and barrier dysfunction continue to impose substantial clinical burdens on affected individuals.^30,31^ Elucidating the biological underpinnings of diarrhea-associated intestinal injury thus holds paramount scientific and translational significance. In this study, we demonstrated that gut microbiota-derived EVs from diarrheal donors induce intestinal inflammatory damage in recipient mice through a gut microbiota-independent mechanism. Notably, EVs-encapsulated miR-125b originating from diarrheal gut microbiota activated pro-inflammatory macrophage polarization, consequently compromising epithelial barrier integrity. These findings collectively suggest that targeted modulation of EVs-mediated inflammatory cascades may represent a novel therapeutic paradigm for mitigating diarrhea-induced intestinal pathology.

Swine are emerging as compelling and physiologically relevant biomedical models for disease investigation due to their conserved metabolic and genetic homology with humans.^32^ Of particular translational significance, the high incidence rates of diarrhea in post-weaning piglets, mirroring the clinical context where weaning-induced diarrhea constitutes a major pediatric health concern, establish this model as a robust platform for dissecting diarrheal pathogenesis.^33,34^ Gut microbial dysbiosis has been established as a critical causative agent in the pathogenesis of diarrhea and intestinal inflammatory damage.^35,36^ Previous study has demonstrated that fecal microbiota transplantation effectively reduces diarrhea incidence in weaned piglets, substantiating the microbiota’s protective role.^37^ Intriguingly, even heat- inactivated fecal suspensions from healthy donors retain therapeutic efficacy, suggesting that non-viable microbial components, particularly bioactive derivatives, may mediate these beneficial effects.^38^ EVs serve as critical mediators of intercellular communication by trafficking molecular cargoes across cellular microenvironments.^36,39^ The budding process of gut microbiota-derived EVs and their subsequent systemic dissemination via host circulatory pathways have been extensively documented.^40^ Elevated EVs biogenesis from gut microbiota has been posited to result from heightened microbial stress responses within the intestinal milieu.^41^ In the present study, we revealed no discernible difference in EVs production between diarrheal piglets and healthy controls. Given that EVs functionality is principally dictated by cargo composition rather than quantity,^42,43^ we hypothesize pathogenicity-specific disparities in EVs molecular payloads between diarrheal and healthy gut microbiota-derived EVs.

Mounting evidence demonstrated that EVs-encapsulated molecular cargos exert profound impacts on tissue inflammation and intestinal pathogenesis.^44,45^ Pertinent study demonstrated that gut microbiota-derived EVs can translocate across the intestinal epithelial barrier, subsequently migrating to distal organs or interfacing with host immune components.^46,47^ Our data revealed that EVs originating from diarrheal piglet gut microbiota induce murine intestinal inflammatory damage. Existing paradigms suggested bacterial EVs-mediated immunomodulation partially depends on microbiota regulation.^48^ Our study demonstrated that EVs-driven inflammatory pathogenesis occurs independent of live microbiota involvement, a finding corroborating recent breakthroughs.^49^ Mammalian macrophages phagocytically internalize EVs, enabling cytoplasmic delivery of bioactive payloads that reprogram immunometabolic functions.^50^ This establishes EVs as pivotal intermediaries in macrophage polarization, whereby direct EVs-macrophage interactions govern inflammatory plasticity and intestinal inflammatory cascades. In this study, we observed EVs accumulation within macrophages. Furthermore, both *in vitro* and *in vivo* models conclusively show that gut microbiota-derived EVs enhance pro-inflammatory macrophage polarization and compromise epithelial barrier integrity through microbiota-independent pathways. These findings expand the conceptual framework of gut microbiota regulation in diarrheal pathology by introducing EVs-mediated trans-kingdom signaling. Nevertheless, the mechanistic specifics whereby EVs molecular constituents from diarrheal microbiota orchestrate intestinal homeostasis warrant systematic elucidation.

The NF-κB signaling pathway emerges as a pivotal driver of pro-inflammatory cytokine expression in macrophages following pathogenic stimulation.^51^ Activated NF-κB signaling orchestrates macrophage polarization toward pro-inflammatory phenotypes via metabolic reprogramming.^52^ Consistently, our study revealed that EVs from diarrheal microbiota promote pro-inflammatory macrophage polarization by augmenting NF-κB protein expression, while exhibiting diminished regulatory capacity in NF-κB-deficient macrophages. Current research suggested predominantly focus on proteomic characterization of microbial EVs, with limited exploration of nucleic acid constituents.^53^ Our data unveiled substantial disparities in miRNA profiles between EVs derived from diarrheal versus healthy piglet. Recent report implicated miR-125b as a critical mediator initiating inflammatory cascades through NF-κB-mediated pro-inflammatory macrophage polarization, ultimately precipitating tissue damage.^54,55^ Notably, we observed significant enrichment of EVs-encapsulated miR-125b in diarrheal microbiota. Furthermore, we performed *in vivo* and *in vitro* experiments to demonstrated that miR-125b disrupts epithelial barrier integrity by activating the NF-κB signaling pathway to drive pro-inflammatory macrophage polarization.

We further elucidated the mechanistic basis through which gut microbiota-derived EVs instigate diarrhea-associated intestinal inflammatory injury. As pivotal functional constituents of extracellular vesicles, miRNAs orchestrate gene regulation through assembly of miRNA-guided silencing complexes that target specific mRNAs, exerting multi-layered control spanning transcriptional and post-transcriptional processes.^56,57^ The NF-κBIA-encoded protein functions as a transcriptional regulator that sequesters NF-κB in the cytoplasm, thereby blocking its nuclear translocation and transcriptional activity.^58,59^ In our study, through miRNA target prediction algorithms, NF-κBIA was identified as a putative target of miR-125b, a finding corroborated by *in vivo* experiment demonstrating miR-125b-mediated suppression of NF-κBIA expression. Macrophages, serving as central orchestrators of inflammatory responses, exhibit phenotypic plasticity between pro- and anti-inflammatory states in response to intestinal microenvironmental cues, a dynamic equilibrium critical for gut homeostasis.^60^ In the present study, adoptive transfer of miR-125b-treated primary macrophages into murine models induced marked intestinal barrier dysfunction. These findings delineated a pathogenic axis wherein diarrheal gut microbiota-derived EVs-enriched miR-125b suppresses NF-κBIA expression, activating NF-κB signaling to drive pro-inflammatory macrophage polarization and initiate inflammatory cascades, culminating in gut epithelial barrier compromise.

Profound alterations in gut microbial composition have been shown to trigger compositional remodeling of EVs. While our investigation uncovered the functional contribution of EVs-encapsulated miR-125b to intestinal inflammatory pathology, the biological significance of numerous co-identified miRNAs remains an open question. Equally worth in-depth study is how to completely exclude host-derived contamination in EVs extracted from feces. Additionally, although we have proven the mechanistic actions of EVs, the pathophysiological significance of EVs observed in these murine models necessitates concurrent validation in human clinical cohorts to assess translational implications.

## Conclusions

In conclusion, this study provides the first demonstration of gut microbiota-derived EVs-mediated pathogenesis in diarrhea-associated intestinal inflammatory injury, delineating a tripartite mechanistic axis linking gut microbial dysbiosis, EVs-encapsulated functional miRNAs, and host inflammatory cascades. We found that diarrheal microbiota-derived EVs orchestrate pro-inflammatory macrophage polarization to compromise epithelial barrier integrity. Additionally, miR-125b-enriched EVs isolated from diarrheal donor inhibited NF-κBIA expression in macrophages. This action activates the NF-κB signaling pathway, driving the polarization of pro-inflammatory macrophages. Polarized pro-inflammatory macrophages further contributed to the damage of intestinal barrier function. Overall, our findings broaden the existing mechanistic insights into diarrhea-associated intestinal pathologies while establishing a foundational framework for the rational design of preventive or therapeutic molecular targets.

## Supplementary Material

Supporting_Information.docx

## Data Availability

The datasets supporting the conclusions of this article are available in the NCBI Sequence Read Archive (SRA) repository under accession number PRJNA1243748 and PRJNA1280547 (16S rRNA Sequencing) and PRJNA1243749 (miRNA sequencing).

## References

[1] DuPont HL. Persistent diarrhea: a clinical review. JAMA. 2016;315(24):2712–24. doi: 10.1001/jama.2016.7833. 27357241

[2] He Z, Ma Y, Yang S, Zhang S, Liu S, Xiao J, Wang Y, Wang W, Yang H, Li S, et al. Gut microbiota-derived ursodeoxycholic acid from neonatal dairy calves improves intestinal homeostasis and colitis to attenuate extended-spectrum beta-lactamase-producing enteroaggregative Escherichia coli infection. Microbiome. 2022;10(1):79. doi:10.1186/s40168-022-01269-0. 35643532 PMC9142728

[3] John CC, Black MM, Nelson CA. Neurodevelopment: the impact of nutrition and inflammation during Early to middle childhood in low-resource settings. Pediatrics. 2017;139(Supplement_1):S59–S71. doi:10.1542/peds.2016-2828H. 28562249 PMC5694688

[4] Gao J, Xiong T, Grabauskas G, Owyang C. Mucosal serotonin reuptake transporter expression in irritable bowel syndrome is modulated by gut microbiota via mast cell-prostaglandin E2. Gastroenterology. 2022;162(7):1962–74 e6. doi:10.1053/j.gastro.2022.02.016. 35167867 PMC9117493

[5] Paulson KR, Kamath AM, Alam T, Bienhoff K, Abady GG, Abbas J, Abbasi-Kangevari M, Abbastabar H, Abd-Allah F, Abd-Elsalam SM, et al. Global, regional, and national progress towards sustainable development goal 3.2 for neonatal and child health: all-cause and cause-specific mortality findings from the global burden of disease study 2019. The Lancet. 2021;398(10303):870–905. doi:10.1016/S0140-6736(21)01207-1. PMC8429803 34416195

[6] Thiagarajah JR, Donowitz M, Verkman AS. Secretory diarrhoea: mechanisms and emerging therapies. Nat Rev Gastroenterol Hepatol. 2015;12(8):446–457. doi:10.1038/nrgastro.2015.111. 26122478 PMC4786374

[7] Young VB, Schmidt TM. Antibiotic-associated diarrhea accompanied by large-scale alterations in the composition of the fecal microbiota. J Clin Microbiol. 2004;42(3):1203–1206. doi:10.1128/JCM.42.3.1203-1206.2004. 15004076 PMC356823

[8] Carroll IM, Ringel-Kulka T, Siddle JP, Ringel Y. Alterations in composition and diversity of the intestinal microbiota in patients with diarrhea-predominant irritable bowel syndrome. Neurogastroenterology Motil. 2012;24(6):521–30, e248. doi:10.1111/j.1365-2982.2012.01891.x. PMC3975596 22339879

[9] Tao S, Fan J, Li J, Wu Z, Yao Y, Wang Z, Wu Y, Liu X, Xiao Y, Wei H, et al. Extracellular vesicles derived from Lactobacillus johnsonii promote gut barrier homeostasis by enhancing M2 macrophage polarization. J Adv Res. 2025;69:545–563. doi: 10.1016/j.jare.2024.03.011. 38508446 PMC11954842

[10] Li J, Feng S, Wang Z, He J, Zhang Z, Zou H, Wu Z, Liu X, Wei H, Tao S. Limosilactobacillus mucosae-derived extracellular vesicles modulates macrophage phenotype and orchestrates gut homeostasis in a diarrheal piglet model. Npj Biofilms Microbiomes. 2023;9(1):33. doi:10.1038/s41522-023-00403-6. 37280255 PMC10244441

[11] Ashida H, Ogawa M, Kim M, Mimuro H, Sasakawa C. Bacteria and host interactions in the gut epithelial barrier. Nat Chem Biol. 2011;8(1):36–45. doi:10.1038/nchembio.741. 22173358

[12] Schwechheimer C, Kuehn MJ. Outer-membrane vesicles from gram-negative bacteria: biogenesis and functions. Nat Rev Microbiol. 2015;13(10):605–619. doi: 10.1038/nrmicro3525. 26373371 PMC5308417

[13] Kalluri R, LeBleu VS. The biology, function, and biomedical applications of exosomes. Science. 2020;367(6478). doi:10.1126/science.aau6977. PMC7717626 32029601

[14] Hu X, Guo J, Zhao C, Jiang P, Maimai T, Yanyi L, Cao Y, Fu Y, Zhang N. The gut microbiota contributes to the development of *Staphylococcus aureus*-induced mastitis in mice. The ISME J. 2020;14(7):1897–1910. doi:10.1038/s41396-020-0651-1. 32341472 PMC7305118

[15] Qiu M, Ye C, Zhao X, Zou C, Tang R, Xie J, Liu Y, Hu Y, Hu X, Zhang N, et al. Succinate exacerbates mastitis in mice via extracellular vesicles derived from the gut microbiota: a potential new mechanism for mastitis. J Nanobiotechnol. 2024;22(1):712. doi:10.1186/s12951-024-02997-1. PMC11566393 39543623

[16] Luo Z, Ji Y, Gao H, Gomes Dos Reis FC, Bandyopadhyay G, Jin Z, Ly C, Chang Y-J, Zhang D, Kumar D, et al. Crig(+) macrophages prevent gut microbial DNA-containing extracellular vesicle-induced tissue inflammation and insulin resistance. Gastroenterology. 2021;160(3):863–874. doi:10.1053/j.gastro.2020.10.042. 33152356 PMC7878308

[17] Behrouzi A, Mazaheri H, Falsafi S, Tavassol ZH, Moshiri A, Siadat SD. Intestinal effect of the probiotic Escherichia coli strain Nissle 1917 and its OMV. J Diabetes Metabolic Disord. 2020;19(1):597–604. doi:10.1007/s40200-020-00511-6. PMC7271297 32550212

[18] Auyeung VC, Ulitsky I, McGeary SE, Bartel DP. Beyond secondary structure: primary-sequence determinants license pri-miRNA hairpins for processing. Cell. 2013;152(4):844–858. doi:10.1016/j.cell.2013.01.031. 23415231 PMC3707628

[19] Yu X, Wang QL, Li YF, Wang XD, Xu A, Li Y. A novel miR-200b-3p/p38IP pair regulates monocyte/macrophage differentiation. Cell Discov. 2016;2(1):15043. doi: 10.1038/celldisc.2015.43. 27462440 PMC4860955

[20] Shen Q, Huang Z, Ma L, Yao J, Luo T, Zhao Y, Xiao Y, Jin Y. Extracellular vesicle miRnas promote the intestinal microenvironment by interacting with microbes in colitis. Gut Microbes. 2022;14(1):2128604. doi:10.1080/19490976.2022.2128604. 36176029 PMC9542864

[21] Casado-Bedmar M, Roy M, Berthet L, Hugot JP, Yang C, Manceau H, Peoc’h K, Chassaing B, Merlin D, Viennois E, et al. Fecal let-7b and miR-21 directly modulate the intestinal microbiota, driving chronic inflammation. Gut Microbes. 2024;16(1):2394249. doi:10.1080/19490976.2024.2394249. 39224018 PMC11376420

[22] Hermann-Bank ML, Skovgaard K, Stockmarr A, Strube ML, Larsen N, Kongsted H, Ingerslev H-C, Mølbak L, Boye M. Characterization of the bacterial gut microbiota of piglets suffering from new neonatal porcine diarrhoea. BMC Vet Res. 2015;11(1):139. doi:10.1186/s12917-015-0419-4. 26099928 PMC4476181

[23] Yin J, Li Y, Han H, Chen S, Gao J, Liu G, Wu X, Deng J, Yu Q, Huang X, et al. Melatonin reprogramming of gut microbiota improves lipid dysmetabolism in high-fat diet-fed mice. J Pineal Res. 2018;65(4):e12524. doi:10.1111/jpi.12524. 30230594

[24] Li L, Ma L, Zhao Z, Luo S, Gong B, Li J, Feng J, Zhang H, Qi W, Zhou T, et al. IL-25-induced shifts in macrophage polarization promote development of beige fat and improve metabolic homeostasis in mice. PLOS Biol. 2021;19(8):e3001348. doi:10.1371/journal.pbio.3001348. 34351905 PMC8341513

[25] Bain CC, Jenkins SJ. Isolation and identification of murine serous cavity macrophages. Methods Mol Biol. 2018;1784:51–67. 29761387 10.1007/978-1-4939-7837-3_5

[26] Ma L, Lyu W, Song Y, Chen K, Lv L, Yang H, Wang W, Xiao Y. Anti-inflammatory effect of clostridium butyricum-derived extracellular vesicles in ulcerative colitis: impact on host microRNAs expressions and gut microbiome profiles. Mol Nutr Food Res. 2023;67(13):e2200884. doi:10.1002/mnfr.202200884. 37183784

[27] Peng S, Xu C, He Q, Xu JM, Kiani FA, Choudhary OP, Idrees A, Mares MM, Wu Y, Li K. Fucoidan alleviates intestine damage in mice induced by LPS via regulation of microbiota. Pak Vet J. 2024;44:517–525.

[28] Meng AQ, Zhang XJ, Pubu P, Ali M, Wang J, Xu C, Almutairi MH, Li K. Protective effect of lentinan against LPS-Induced injury in mice via influencing antioxidant enzyme activity, inflammatory pathways and gut microbiota. Pak Vet J. 2024;44:647–656.

[29] da Cruz Gouveia MA, Lins MTC, da Silva GAP. Acute diarrhea with blood: diagnosis and drug treatment. J Pediatr (rio J). 2020;96 Suppl 1:20–28. doi:10.1016/j.jped.2019.08.006. PMC9432323 31604059

[30] Troeger CE, Khalil IA, Blacker BF, Biehl MH, Albertson SB, Zimsen SRM, Rao PC, Abate D, Ahmadi A, Ahmed MLCB, et al. Quantifying risks and interventions that have affected the burden of diarrhoea among children younger than 5 years: an analysis of the global burden of disease study 2017. The Lancet Infectious Diseases. 2020;20(1):37–59. doi:10.1016/S1473-3099(19)30401-3. 31678029 PMC7340495

[31] Troeger C, Colombara DV, Rao PC, Khalil IA, Brown A, Brewer TG, Guerrant RL, Houpt ER, Kotloff KL, Misra K, et al. Global disability-adjusted life-year estimates of long-term health burden and undernutrition attributable to diarrhoeal diseases in children younger than 5 years. Lancet Glob Health. 2018;6(3):e255–e69. doi: 10.1016/S2214-109X(18)30045-7. 29433665 PMC5861379

[32] Lunney JK, Van Goor A, Walker KE, Hailstock T, Franklin J, Dai C. Importance of the pig as a human biomedical model. Sci Transl Med. 2021;13(621):eabd5758. doi:10.1126/scitranslmed.abd5758. 34818055

[33] Ochoa TJ, Chea-Woo E, Baiocchi N, Pecho I, Campos M, Prada A, Valdiviezo G, Lluque A, Lai D, Cleary TG, et al. Randomized double-blind controlled trial of bovine lactoferrin for prevention of diarrhea in children. J Pediatr. 2013;162(2):349–356. doi:10.1016/j.jpeds.2012.07.043. 22939927 PMC3547155

[34] Fawzy A, Arpadi S, Kankasa C, Sinkala M, Mwiya M, Thea DM, Aldrovandi GM, Kuhn L. Early weaning increases diarrhea morbidity and mortality among uninfected children born to HIV-infected mothers in Zambia. The J Infect Dis. 2011;203(9):1222–1230. doi:10.1093/infdis/jir019. 21459815 PMC3069726

[35] Ma X, Zhang Y, Xu T, Qian M, Yang Z, Zhan X, Han X. Early-life intervention using exogenous fecal microbiota alleviates gut injury and reduce inflammation caused by weaning stress in piglets. Front Microbiol. 2021;12:671683. doi: 10.3389/fmicb.2021.671683. 34177852 PMC8222923

[36] Fan J, Zhang Y, Zuo M, Ding S, Li J, Feng S, Xiao Y, Tao S. Novel mechanism by which extracellular vesicles derived from Lactobacillus murinus alleviates deoxynivalenol-induced intestinal barrier disruption. Environ Int. 2024;185:108525. doi:10.1016/j.envint.2024.108525. 38408410

[37] Hu J, Ma L, Nie Y, Chen J, Zheng W, Wang X, Xie C, Zheng Z, Wang Z, Yang T, et al. A microbiota-derived bacteriocin targets the host to confer diarrhea resistance in early-weaned piglets. Cell Host Microbe. 2018;24(6):817–32 e8. doi:10.1016/j.chom.2018.11.006. 30543777

[38] Zhou X, Liu Y, Xiong X, Chen J, Tang W, He L, Zhang Z, Yin Y, Li F. Intestinal accumulation of microbiota-produced succinate caused by loss of microRNAs leads to diarrhea in weanling piglets. Gut Microbes. 2022;14(1):2091369. doi: 10.1080/19490976.2022.2091369. 35758253 PMC9235893

[39] Kim JH, Lee J, Park J, Gho YS. Gram-negative and gram-positive bacterial extracellular vesicles. Semin Cell Dev Biol. 2015;40:97–104. doi: 10.1016/j.semcdb.2015.02.006. 25704309

[40] Chelakkot C, Choi Y, Kim DK, Park HT, Ghim J, Kwon Y, Jeon J, Kim M-S, Jee Y-K, Gho YS, et al. Akkermansia muciniphila-derived extracellular vesicles influence gut permeability through the regulation of tight junctions. Exp Mol Med. 2018;50(2):e450. doi: 10.1038/emm.2017.282. 29472701 PMC5903829

[41] Macia L, Nanan R, Hosseini-Beheshti E, Grau GE. Host- and microbiota-derived extracellular vesicles, immune function, and disease development. IJMS. 2019;21(1):107. doi: 10.3390/ijms21010107. 31877909 PMC6982009

[42] Lener T, Gimona M, Aigner L, Borger V, Buzas E, Camussi G, Chaput N, Chatterjee D, Court FA, Del Portillo HA, et al. Applying extracellular vesicles based therapeutics in clinical trials - an ISEV position paper. J Extracell Vesicles. 2015;4(1):30087. doi: 10.3402/jev.v4.30087. 26725829 PMC4698466

[43] Thery C, Witwer KW, Aikawa E, Alcaraz MJ, Anderson JD, Andriantsitohaina R, Antoniou A, Arab T, Archer F, Atkin‐Smith GK, et al. Minimal information for studies of extracellular vesicles 2018 (MISEV2018): a position statement of the International Society for Extracellular Vesicles and update of the MISEV2014 guidelines. J Extracell Vesicles. 2018;7(1):1535750. doi: 10.1080/20013078.2018.1535750. 30637094 PMC6322352

[44] Mazzariol M, Camussi G, Brizzi MF. Extracellular vesicles tune the immune system in renal disease: a focus on systemic lupus erythematosus, antiphospholipid syndrome, thrombotic microangiopathy and ANCA-Vasculitis. IJMS. 2021;22(8):4194. doi: 10.3390/ijms22084194. 33919576 PMC8073859

[45] Cui L, Yang R, Huo D, Li L, Qu X, Wang J, Wang X, Liu H, Chen H, Wang X, et al. Streptococcus pneumoniae extracellular vesicles aggravate alveolar epithelial barrier disruption via autophagic degradation of OCLN (occludin). Autophagy. 2024;20(7):1577–1596. doi: 10.1080/15548627.2024.2330043. 38497494 PMC11210924

[46] Su D, Li M, Xie Y, Xu Z, Lv G, Jiu Y, Lin J, Chang C-J, Chen H, Cheng F, et al. Gut commensal bacteria parabacteroides goldsteinii-derived outer membrane vesicles suppress skin inflammation in psoriasis. J Control Release. 2025;377:127–145. doi: 10.1016/j.jconrel.2024.11.014. 39532207

[47] Chen Y, Huang X, Liu A, Fan S, Liu S, Li Z, Yang X, Guo H, Wu M, Liu M, et al. Lactobacillus reuteri vesicles regulate mitochondrial function of macrophages to promote mucosal and cutaneous wound healing. Adv Sci (Weinh). 2024;11(24):e2309725. doi: 10.1002/advs.202309725. 38647360 PMC11199966

[48] Wang X, Lin S, Wang L, Cao Z, Zhang M, Zhang Y, Liu R, Liu J. Versatility of bacterial outer membrane vesicles in regulating intestinal homeostasis. Sci Adv. 2023;9(11):eade5079. doi: 10.1126/sciadv.ade5079. 36921043 PMC10017049

[49] Nie X, Li Q, Ji H, Zhang S, Wang Y, Xie J, Nie S. Bifidobacterium longum NSP001-derived extracellular vesicles ameliorate ulcerative colitis by modulating T cell responses in gut microbiota-(in)dependent manners. Npj Biofilms Microbiomes. 2025;11(1):27. doi: 10.1038/s41522-025-00663-4. 39929833 PMC11811157

[50] Diaz-Garrido N, Badia J, Baldoma L. Microbiota-derived extracellular vesicles in interkingdom communication in the gut. J Extracell Vesicles. 2021;10(13):e12161. doi: 10.1002/jev2.12161. 34738337 PMC8568775

[51] Liu L, Guo H, Song A, Huang J, Zhang Y, Jin S, Li S, Zhang L, Yang C, Yang P. Progranulin inhibits LPS-induced macrophage M1 polarization via NF-small ka, CyrillicB and MAPK pathways. BMC Immunol. 2020;21(1):32. doi: 10.1186/s12865-020-00355-y. 32503416 PMC7275413

[52] Sanchez-Rodriguez R, Tezze C, Agnellini AHR, Angioni R, Venegas FC, Cioccarelli C, Munari F, Bertoldi N, Canton M, Desbats MA, et al. OPA1 drives macrophage metabolism and functional commitment via p65 signaling. Cell Death Differ. 2023;30(3):742–752. doi: 10.1038/s41418-022-01076-y. 36307526 PMC9984365

[53] Liang X, Dai N, Sheng K, Lu H, Wang J, Chen L, Wang Y. Gut bacterial extracellular vesicles: important players in regulating intestinal microenvironment. Gut Microbes. 2022;14(1):2134689. doi: 10.1080/19490976.2022.2134689. 36242585 PMC9578468

[54] Essandoh K, Li Y, Huo J, Fan GC. MiRNA-Mediated macrophage polarization and its potential role in the regulation of inflammatory response. Shock. 2016;46(2):122–131. doi: 10.1097/SHK.0000000000000604. 26954942 PMC4949115

[55] Haemmig S, Baumgartner U, Gluck A, Zbinden S, Tschan MP, Kappeler A, Mariani L, Vajtai I, Vajtai E. miR-125b controls apoptosis and temozolomide resistance by targeting TNFAIP3 and NKIRAS2 in glioblastomas. Cell Death Dis. 2014;5(6):e1279. doi: 10.1038/cddis.2014.245. 24901050 PMC4611719

[56] Nobrega M, Reis MBD, Souza MF, Furini HH, Costa Brandao Berti F, Souza ILM, Mingorance Carvalho T, Zanata SM, Fuganti PE, Malheiros D, et al. Comparative analysis of extracellular vesicles miRnas (EV-miRnas) and cell-free microRNAs (cf-miRnas) reveals that EV-miRnas are more promising as diagnostic and prognostic biomarkers for prostate cancer. Gene. 2025;939:149186. doi: 10.1016/j.gene.2024.149186. 39708932

[57] Ji H, Chen M, Greening DW, He W, Rai A, Zhang W, Simpson RJ. Deep sequencing of RNA from three different extracellular vesicle (EV) subtypes released from the human LIM1863 colon cancer cell line uncovers distinct miRNA-enrichment signatures. PLOS ONE. 2014;9(10):e110314. doi: 10.1371/journal.pone.0110314. 25330373 PMC4201526

[58] Wang M, Li Q, Ren B, Hao D, Guo H, Yang L, Wang Z, Dai L. Ethanolic extract of Arctium lappa leaves alleviates cerebral ischemia reperfusion-induced inflammatory injury via HDAC9-mediated NF-kappaB pathway. Phytomedicine. 2024;129:155599. doi: 10.1016/j.phymed.2024.155599. 38669967

[59] Deng C, Huo M, Chu H, Zhuang X, Deng G, Li W, Wei H, Zeng L, He Y, Liu H, et al. Exosome circATP8A1 induces macrophage M2 polarization by regulating the miR-1-3p/STAT6 axis to promote gastric cancer progression. Mol Cancer. 2024;23(1):49. doi:10.1186/s12943-024-01966-4. 38459596 PMC10921793

[60] Han D, Lu D, Huang S, Pang J, Wu Y, Hu J, Zhang X, Pi Y, Zhang G, Wang J, et al. Small extracellular vesicles from Ptpn1-deficient macrophages alleviate intestinal inflammation by reprogramming macrophage polarization via lactadherin enrichment. Redox Biol. 2022;58:102558. doi:10.1016/j.redox.2022.102558. 36462232 PMC9712762

