# Supplementary material for: Diarrheal microbiota-derived extracellular vesicles drive intestinal homeostasis dysfunction via miR-125b/NF-κB-mediated macrophage polarization
Source: Gut Microbes. 2025 Jul 30;17(1):2541036. doi: 10.1080/19490976.2025.2541036 (PMC12320886; doi:10.1080/19490976.2025.2541036)
Supplement: Supporting_Information.docx [file KGMI_A_2541036_SM3911.docx]

**Supporting information**

**Diarrheal microbiota-derived extracellular vesicles drive intestinal homeostasis dysfunction via miR-125b/NF-κB-mediated macrophage polarization**

Mengzhen Song, Wenjun Zhou, Jinping Fan, Chenhao Jia, Wen Xiong, Hong Wei, Shiyu Tao*

Running title: Gut EVs miR-125b disrupts intestinal homeostasis

Authors and Affiliations:

College of Animal Sciences and Technology, Huazhong Agricultural University, Wuhan, China 430070

***Address for reprint requests and other correspondence:**

**Shiyu Tao,** College of Animal Sciences and Technology, Huazhong Agricultural University, Wuhan, China 430070 (e-mail: [sytao@mail.hzau.edu.cn](mailto:sytao@mail.hzau.edu.cn)).

**Table S1:** The synthesis information of mimics, inhibitor and agomiR

| Name | Sequence (5'-3') | Modification |
| --- | --- | --- |
| miR-125b-mimics sense | UCCCUGAGACCCUAACUUGUGA | Not Applicable |
| miR-125b-mimics antisense | ACAAGUUAGGGUCUCAGGGAUU | Not Applicable |
| Mimics-NC sense | UCACAACCUCCUAGAAAGAGUAGA | Not Applicable |
| Mimics-NC antisense | UCACAACCUCCUAGAAAGAGUAGA | Not Applicable |
| miR-125b inhibitors | UCACAAGUUAGGGUCUCAGGGA | Not Applicable |
| agomiR-125b sense | UCCCUGAGACCCUAACUUGUGA | Not Applicable |
| agomiR-125b antisense | (mA)*(mC)*(mA)(mA)(mG)(mU)(mU)(mA)(mG)(mG)(mG)(mU)(mC)(mU)(mC)(mA)(mG)(mG)*(mG)*(mA)*(mU)*(mU) | (mA)'6,(mC)'3,(mG)'7,(mU)'6,3'Cholesteryl,*'6 |
| agomiR-NC sense | UCACAACCUCCUAGAAAGAGUAGA | Not Applicable |
| agomiR-NC antisense | (mU)*(mC)*(mU)(mA)(mC)(mU)(mC)(mU)(mU)(mU)(mC)(mU)(mA)(mG)(mG)(mA)(mG)(mG)(mU)(mU)*(mG)*(mU)*(mG)*(mA) | (mA)'4,(mC)'4,(mG)'6,(mU)'10,3'Cholesteryl,*'6 |

**Table S2:** Primer sequences used in RT-qPCR

| Target genes | Forward sequence (5’→3’) | Reverse sequence (5’→3’) |
| --- | --- | --- |
| IL-1β | CTCGCAGCAGCACATCAACAAG | GGAAGGTCCACGGGAAAGACAC |
| IL-6 | ACCACGGCCTTCCCTACTT | CACAACTCTTTTCTCATTTCCAC |
| IL-10 | CCCTTTGCTATGGTGTCCTT | TGGTTTCTCTTCCCAAGACC |
| TNF-α | TGGGAGTAGACAAGGTACAACCC | CATCTTCTCAAAATTCGAGTGACAA |
| NF-κBIA | TGAAGGACGAGGAGTACGAGC | TTCGTGGATGATTGCCAAGTG |
| β-actin | CATTGCTGACAGGATGCAGAAGG | TGCTGGAAGGTGGACAGTGAGG |

**Table S3:** List of antibodies used for western blot analysis

| Antibodies | Source | Product number | Dilution |
| --- | --- | --- | --- |
| iNOS | Abcam | ab178945 | 1: 1000 |
| Arg1 | Proteitech | 16001 | 1: 5000 |
| p-NF-κB p65 | CST | 3033 | 1: 1000 |
| NF-κB p65 | CST | 8242 | 1: 1000 |
| ZO-1 | Abcam | ab96587 | 1: 1000 |
| Occludin | Abcam | ab167161 | 1: 1000 |
| β-actin | CST | 4967 | 1: 1000 |
| Goat anti-rabbit | Santa Cruz | sc-2004 | 1: 10000 |

**Table S4:** Differentially expressed miRNAs in D-EVs vs. H-EVs

| miRNAs | C-EVs (Mean) | D-EVs (Mean) | Log2FC (D-EVs/C-EVs) | P value | P adjust | Regulate |
| --- | --- | --- | --- | --- | --- | --- |
| ssc-let-7a | 4787.26 | 18946.82 | 2.009 | 0.0000 | 0.0000 | up |
| ssc-let-7c | 2494.50 | 8805.84 | 1.821 | 0.0000 | 0.0000 | up |
| ssc-let-7f-5p | 4339.91 | 14661.59 | 1.755 | 0.0000 | 0.0000 | up |
| ssc-let-7g | 545.90 | 2594.94 | 2.236 | 0.0000 | 0.0000 | up |
| ssc-let-7i-5p | 3057.41 | 7057.73 | 1.252 | 0.0000 | 0.0000 | up |
| ssc-miR-101 | 4349.90 | 16860.43 | 1.977 | 0.0000 | 0.0000 | up |
| ssc-miR-103 | 982.09 | 3594.24 | 1.885 | 0.0000 | 0.0000 | up |
| ssc-miR-122-5p | 54861.85 | 238269.97 | 2.138 | 0.0000 | 0.0000 | up |
| ssc-miR-125a | 1355.07 | 4836.70 | 1.836 | 0.0000 | 0.0000 | up |
| ssc-miR-125b | 2175.54 | 8627.78 | 1.966 | 0.0000 | 0.0000 | up |
| ssc-miR-126-3p | 11398.78 | 40910.33 | 1.809 | 0.0000 | 0.0000 | up |
| ssc-miR-126-5p | 2859.82 | 6938.23 | 1.211 | 0.0000 | 0.0000 | up |
| ssc-miR-1285 | 69.19 | 798.80 | 3.573 | 0.0003 | 0.0009 | up |
| ssc-miR-140-3p | 101.52 | 1263.93 | 3.573 | 0.0000 | 0.0000 | up |
| ssc-miR-142-5p | 519.15 | 2905.19 | 2.474 | 0.0000 | 0.0000 | up |
| ssc-miR-143-3p | 2598.36 | 8471.69 | 1.695 | 0.0000 | 0.0000 | up |
| ssc-miR-1468 | 136.18 | 888.07 | 2.796 | 0.0004 | 0.0013 | up |
| ssc-miR-146a-5p | 246.79 | 2263.23 | 3.269 | 0.0000 | 0.0000 | up |
| ssc-miR-148a-3p | 4533.34 | 18391.44 | 2.042 | 0.0000 | 0.0000 | up |
| ssc-miR-151-3p | 69.19 | 1506.14 | 4.310 | 0.0000 | 0.0000 | up |
| ssc-miR-152 | 308.06 | 1706.87 | 2.404 | 0.0000 | 0.0001 | up |
| ssc-miR-16 | 5882.71 | 37370.02 | 2.587 | 0.0000 | 0.0000 | up |
| ssc-miR-191 | 1071.80 | 7190.18 | 2.816 | 0.0000 | 0.0000 | up |
| ssc-miR-192 | 3429.08 | 22718.62 | 2.754 | 0.0000 | 0.0000 | up |
| ssc-miR-194a-5p | 2045.97 | 9604.15 | 2.231 | 0.0000 | 0.0000 | up |
| ssc-miR-199a-3p | 824.88 | 3906.44 | 2.310 | 0.0000 | 0.0000 | up |
| ssc-miR-199b-3p | 824.88 | 3906.44 | 2.310 | 0.0000 | 0.0000 | up |
| ssc-miR-21-5p | 3570.59 | 18213.39 | 2.362 | 0.0000 | 0.0000 | up |
| ssc-miR-221-3p | 165.13 | 1086.86 | 2.667 | 0.0002 | 0.0008 | up |
| ssc-miR-223 | 848.25 | 6387.97 | 2.899 | 0.0000 | 0.0000 | up |
| ssc-miR-23a | 196.29 | 1221.98 | 2.725 | 0.0000 | 0.0001 | up |
| ssc-miR-23b | 98.14 | 1064.91 | 3.404 | 0.0000 | 0.0001 | up |
| ssc-miR-26a | 2921.75 | 8629.73 | 1.579 | 0.0000 | 0.0000 | up |
| ssc-miR-26b-5p | 2634.59 | 8583.63 | 1.699 | 0.0000 | 0.0000 | up |
| ssc-miR-27a | 206.55 | 1508.09 | 2.863 | 0.0000 | 0.0000 | up |
| ssc-miR-27b-3p | 1353.12 | 3685.70 | 1.561 | 0.0000 | 0.0000 | up |
| ssc-miR-29a-3p | 2822.69 | 13242.53 | 2.228 | 0.0000 | 0.0000 | up |
| ssc-miR-29c | 1634.96 | 4369.14 | 1.404 | 0.0000 | 0.0000 | up |
| ssc-miR-30a-5p | 1052.59 | 3417.89 | 1.735 | 0.0000 | 0.0000 | up |
| ssc-miR-30c-5p | 683.12 | 2396.16 | 1.836 | 0.0000 | 0.0000 | up |
| ssc-miR-30d | 1275.87 | 5346.48 | 2.101 | 0.0000 | 0.0000 | up |
| ssc-miR-30e-5p | 1276.66 | 3548.63 | 1.441 | 0.0000 | 0.0000 | up |
| ssc-miR-335 | 33.49 | 709.29 | 4.310 | 0.0004 | 0.0013 | up |
| ssc-miR-34a | 199.80 | 1551.75 | 2.863 | 0.0000 | 0.0000 | up |
| ssc-miR-423-5p | 33.49 | 621.72 | 4.310 | 0.0004 | 0.0013 | up |
| ssc-miR-451 | 70.36 | 1130.52 | 3.988 | 0.0000 | 0.0000 | up |
| ssc-miR-486 | 556.02 | 3059.35 | 2.448 | 0.0000 | 0.0000 | up |
| ssc-miR-92a | 168.51 | 1506.14 | 2.988 | 0.0000 | 0.0000 | up |
| 1_1492 | 3139.19 | 12188.84 | 2.066 | 0.0000 | 0.0000 | up |
| 10_19352 | 32.32 | 11645.90 | 8.415 | 0.0000 | 0.0000 | up |
| 13_23538 | 32.32 | 2239.33 | 5.988 | 0.0000 | 0.0000 | up |
| 14_25353 | 30676.70 | 74688.51 | 1.231 | 0.0000 | 0.0000 | up |
| 14_25880 | 3126.86 | 1042.95 | -1.519 | 0.0000 | 0.0001 | down |
| 14_26157 | 206.55 | 25782.63 | 6.919 | 0.0000 | 0.0000 | up |
| 14_26575 | 0.00 | 1021.24 | 5.988 | 0.0000 | 0.0000 | up |
| 16_29769 | 0.00 | 2837.63 | 7.310 | 0.0000 | 0.0000 | up |
| 17_30731 | 1617.43 | 111.22 | -3.566 | 0.0000 | 0.0000 | down |
| 18_31475 | 7307.21 | 42891.61 | 2.555 | 0.0000 | 0.0000 | up |
| 2_4139 | 752474.55 | 115491.50 | -2.668 | 0.0000 | 0.0000 | down |
| 3_6481 | 1678.84 | 7893.87 | 2.207 | 0.0000 | 0.0000 | up |
| 3_6873 | 5560.35 | 1219.79 | -2.181 | 0.0000 | 0.0000 | down |
| 3_7909 | 3602.39 | 1043.20 | -1.712 | 0.0000 | 0.0000 | down |
| 6_11928 | 69.19 | 11748.60 | 7.364 | 0.0000 | 0.0000 | up |
| 6_12809 | 873.18 | 0.00 | -3.655 | 0.0002 | 0.0005 | down |
| 7_14033 | 3191.11 | 176.83 | -4.551 | 0.0000 | 0.0000 | down |
| 9_17983 | 36.87 | 1154.18 | 5.158 | 0.0000 | 0.0000 | up |
| 9_18439 | 1122.82 | 9134.86 | 2.966 | 0.0000 | 0.0000 | up |
| AEMK02000452.1_33609 | 513.44 | 3018.36 | 2.605 | 0.0000 | 0.0000 | up |
| X_32086 | 1290.54 | 16920.20 | 3.722 | 0.0000 | 0.0000 | up |
| X_32444 | 0.00 | 2904.71 | 7.381 | 0.0000 | 0.0000 | up |


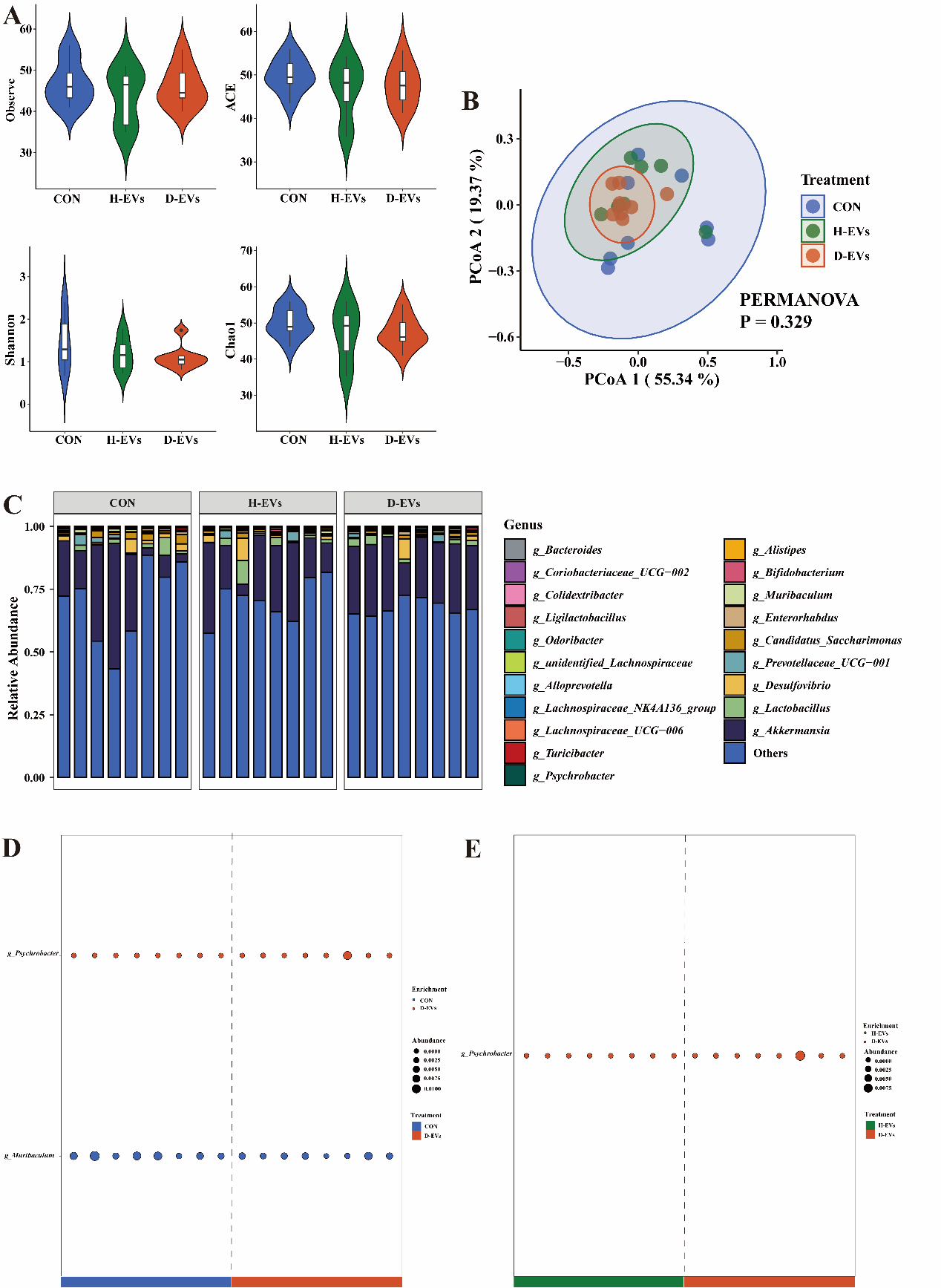


**Figure S1: Fecal microbiota analysis in SPF mice**

(A) α-diversity of fecal microbiota. (B) β-diversity (PCoA) of fecal microbiota. (C) Genus-level taxonomic composition. (D-E) Differential abundance analysis of bacterial genera. n = 8.


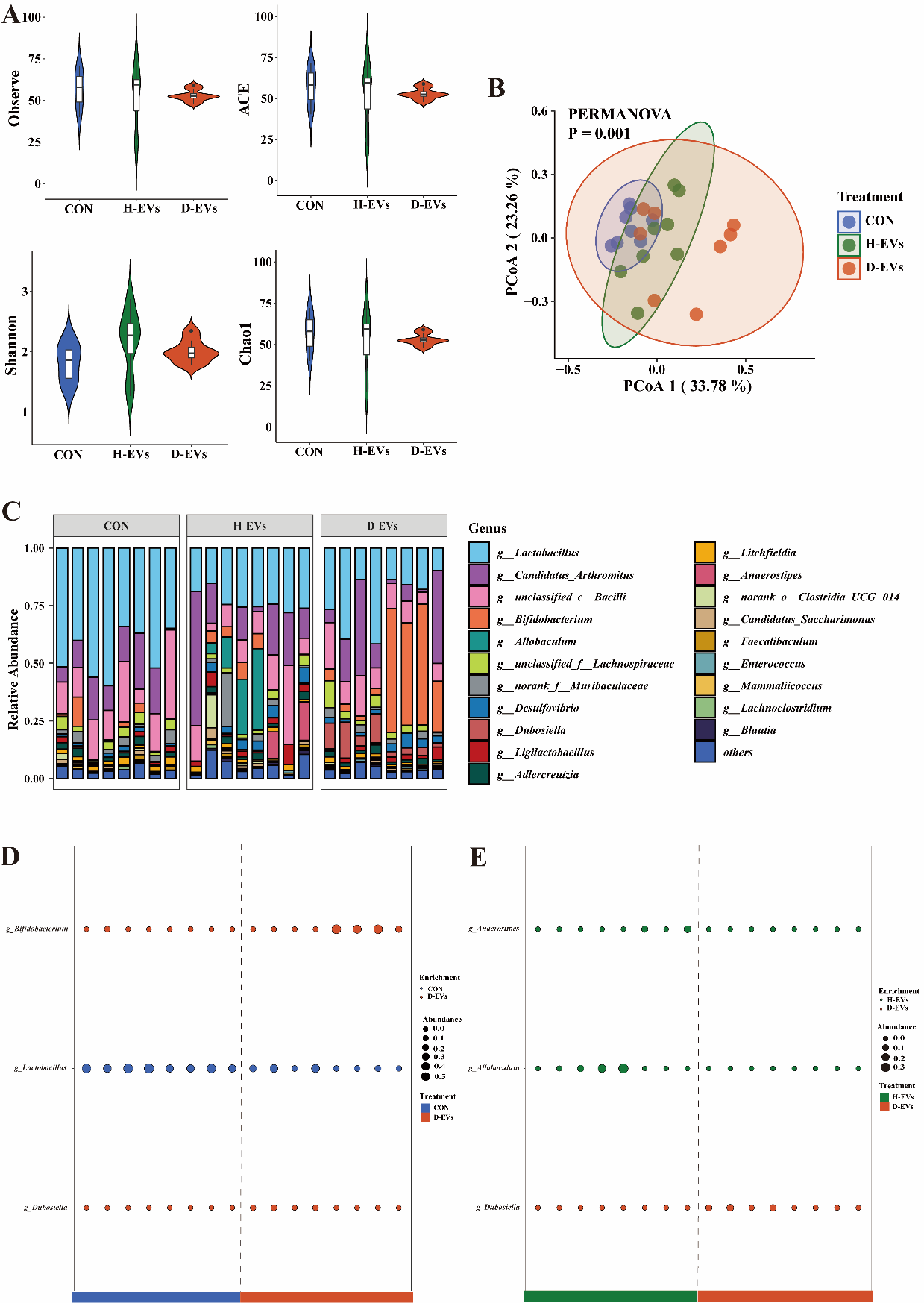


**Figure S2:** **Jejunal microbiota analysis in SPF mice**

(A) α-diversity of jejunal microbiota. (B) β-diversity (PCoA) of jejunal microbiota. (C) Genus-level taxonomic composition. (D-E) Differential abundance analysis of bacterial genera. n = 8.


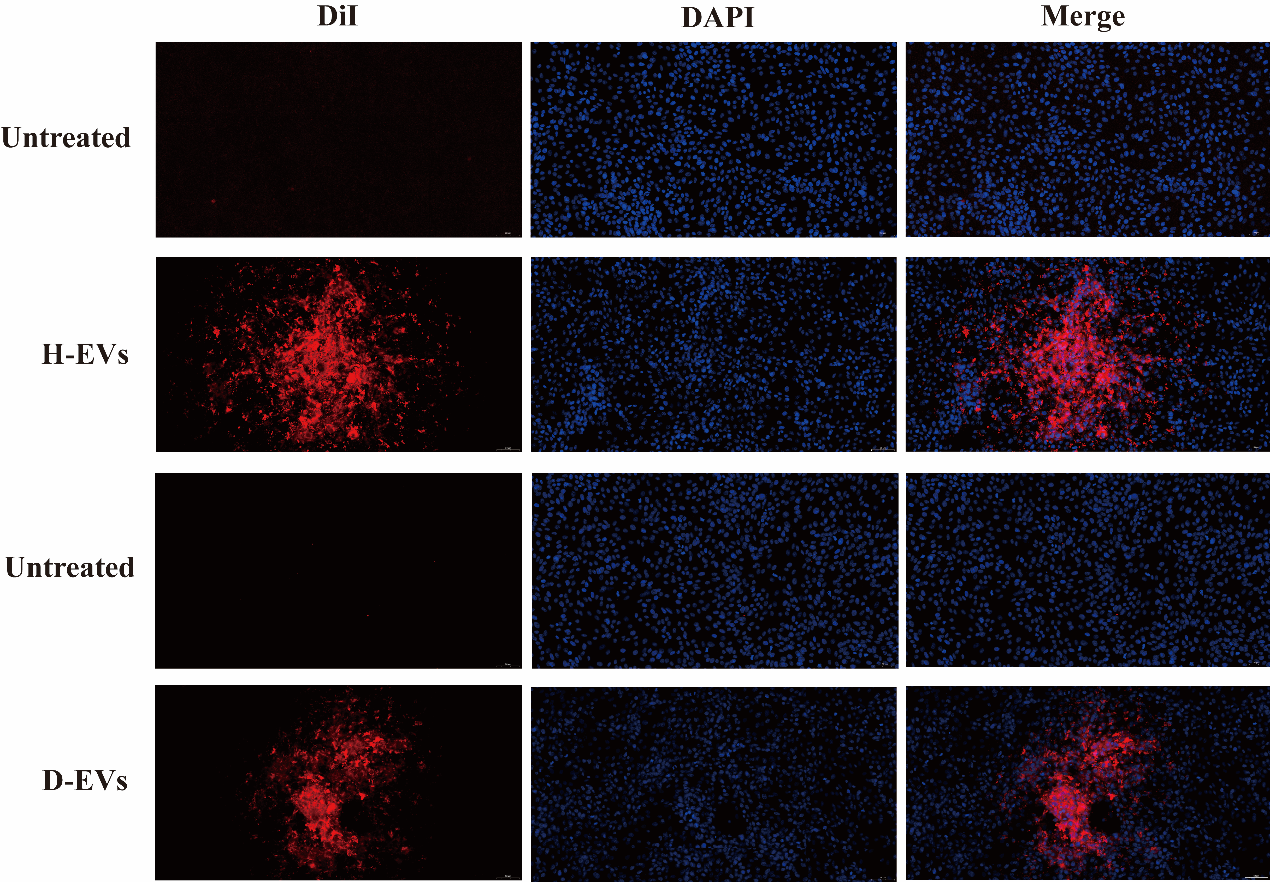


**Figure S3: Fluorescent pictures of EVs endocytosed by macrophages.**


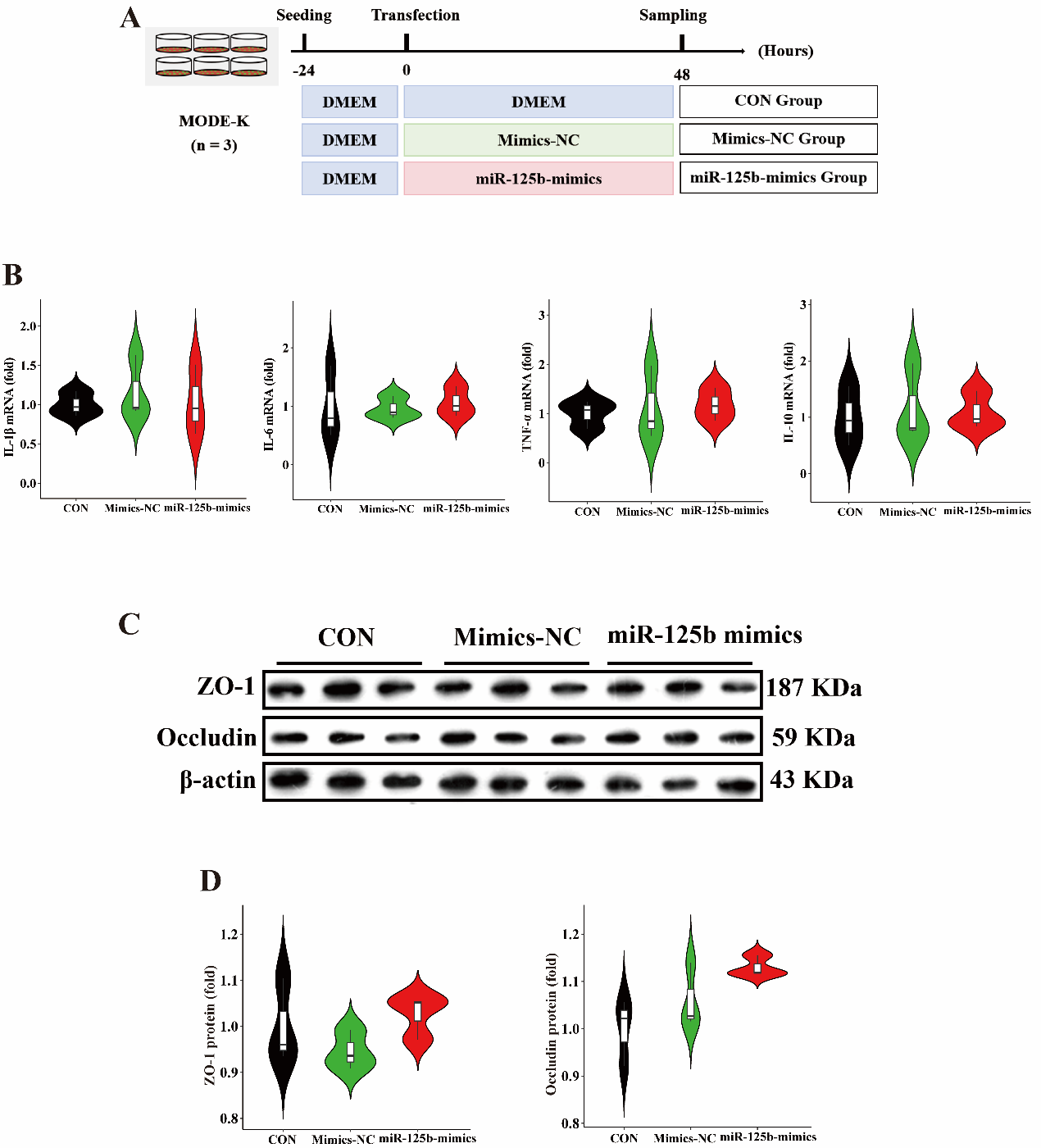


**Figure S4:** **The direct effects of** **miR-125b on intestinal epithelial cells *in vitro***

(A) Experimental workflow. (B) qPCR analysis of IL-1β, IL-6, TNF-α, and IL-10 mRNA in miR-125b-treated intestinal epithelial cells. (C-D) Western blot analysis of tight junction proteins (ZO-1, Occludin). n = 3. Statistical differences were assessed using one-way ANOVA with Tukey's post hoc comparison, with data presented as mean values ± standard error of the mean.


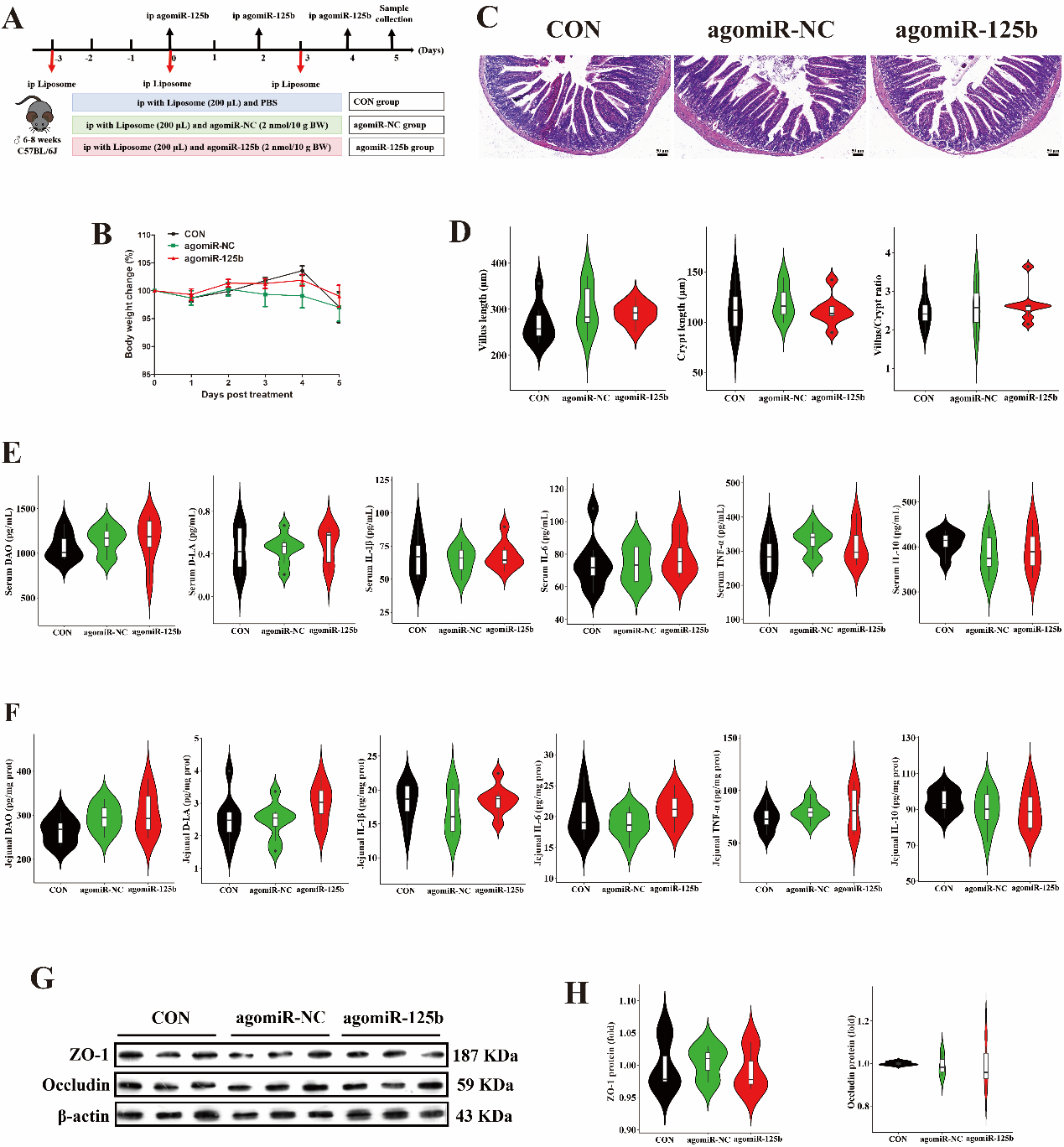


**Figure S5:** **Overexpression of miR-125b fails to induce the pro-inflammatory response and intestinal barrier dysfunction in macrophage-depleted mice**

(A) Experimental workflow. (B) Longitudinal monitoring of animal body mass variations. (C-D) Histopathological evaluation (scale bar, 50 μm) and morphometric analysis of jejunal epithelium using hematoxylin-eosin staining. (E-F) Quantitative determination of intestinal permeability biomarkers (diamine oxidase, D-lactate) and pro-/anti-inflammatory cytokines (IL-1β, IL-6, TNF-α, IL-10) in systemic circulation and local tissue. (G-H) Tight junction protein expression profiling (zona occludens-1, occludin) in intestinal epithelium. Experimental groups comprised 8 biological replicates for physiological and cytokine measurements, and 3 technical replicates for immunoblot analyses. Statistical differences were assessed using one-way ANOVA with Tukey's post hoc comparison, with data presented as mean values ± standard error of the mean.


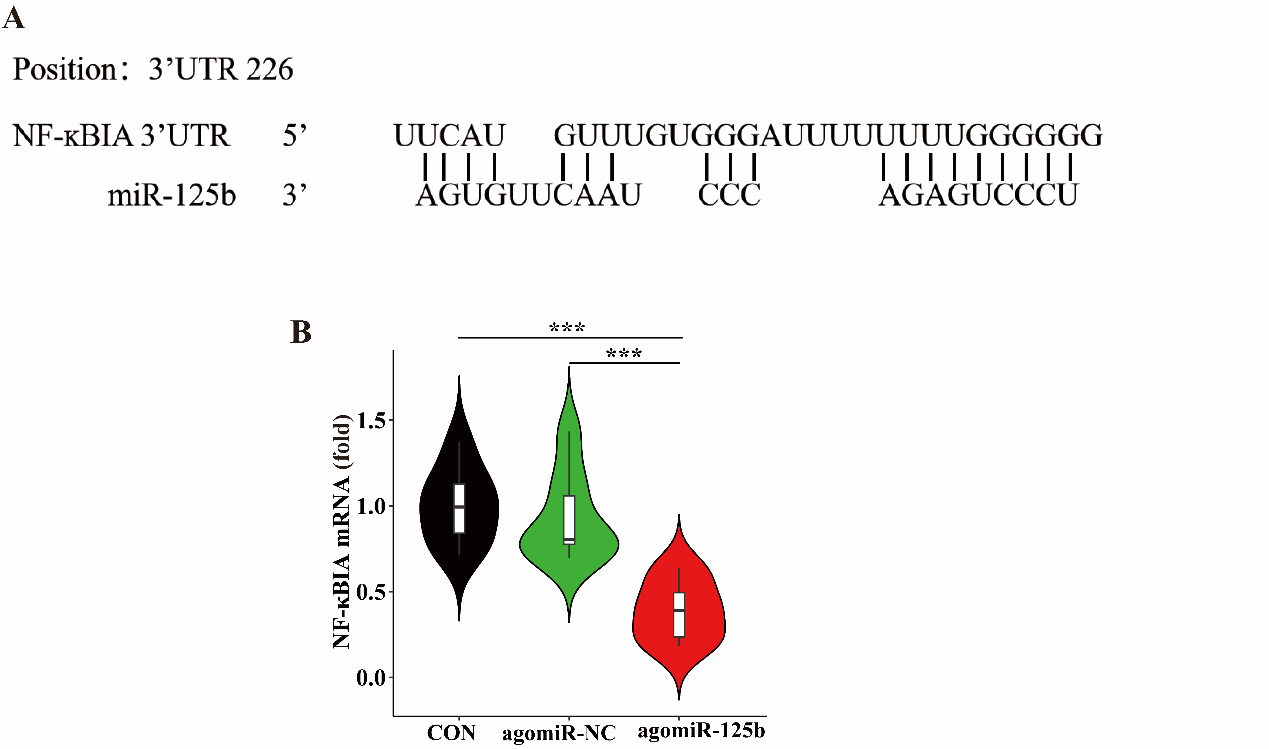


**Figure S6: miR-125b targets NF-κBIA**

(A) Predicted miR-125b binding site in NF-κBIA 3' UTR. (B) qPCR analysis of NF-κBIA mRNA in agomiR-125b-treated jejunum. n = 8. Statistical differences were assessed using one-way ANOVA with Tukey's post hoc comparison, with data presented as mean values ± standard error of the mean. Significance thresholds: ***P < 0.001.


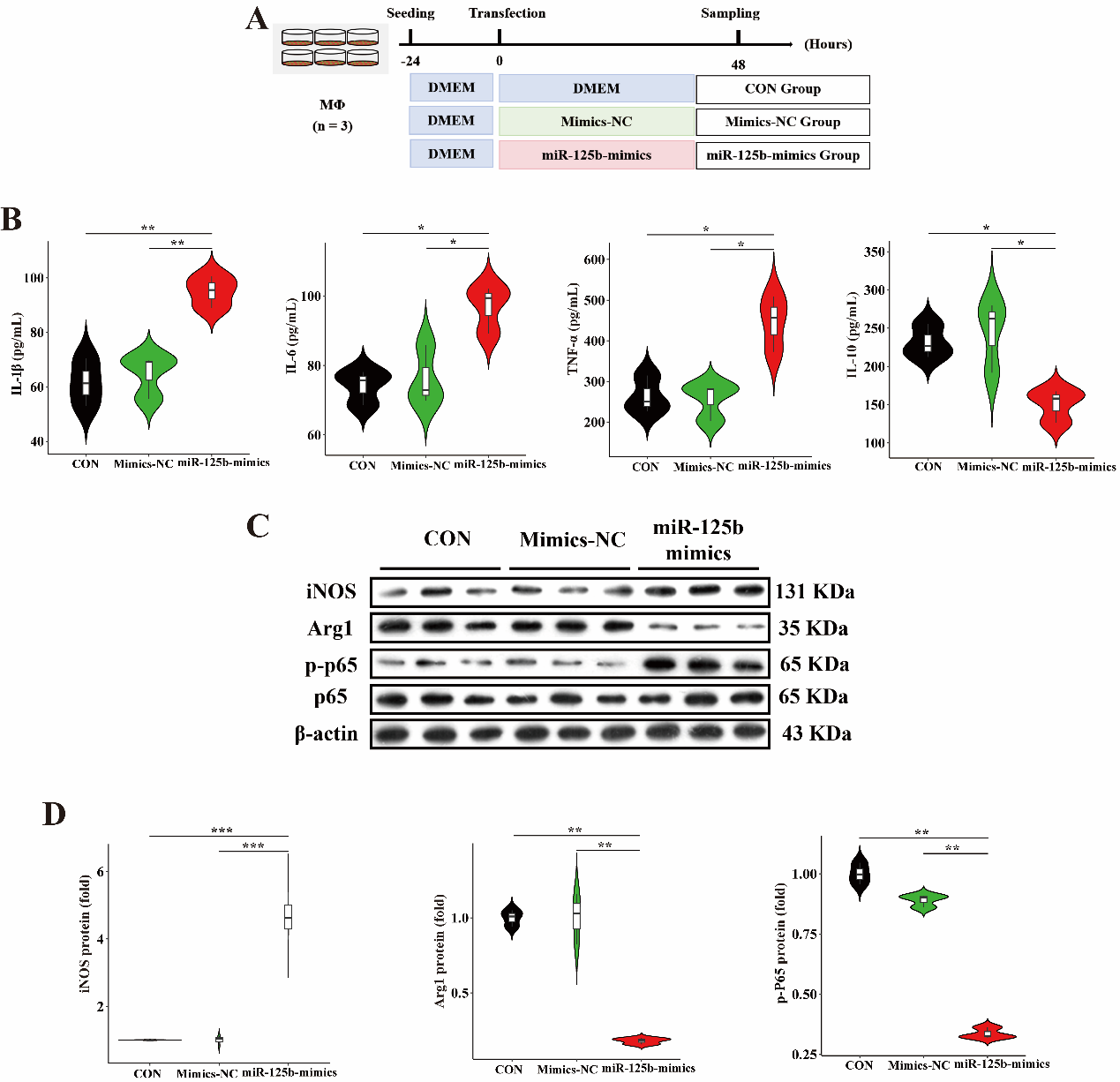


**Figure S7: miR-125b activates primary macrophages**

(A) Experimental workflow. (B) Cytokine levels (IL-1β, IL-6, TNF-α, IL-10) in macrophage supernatants. (C-D) Western blot analysis of macrophage polarization markers (iNOS, Arg1) and NF-κB p65. n = 3. Statistical differences were assessed using one-way ANOVA with Tukey's post hoc comparison, with data presented as mean values ± standard error of the mean. Significance thresholds: *P < 0.05, **P < 0.01, ***P < 0.001.


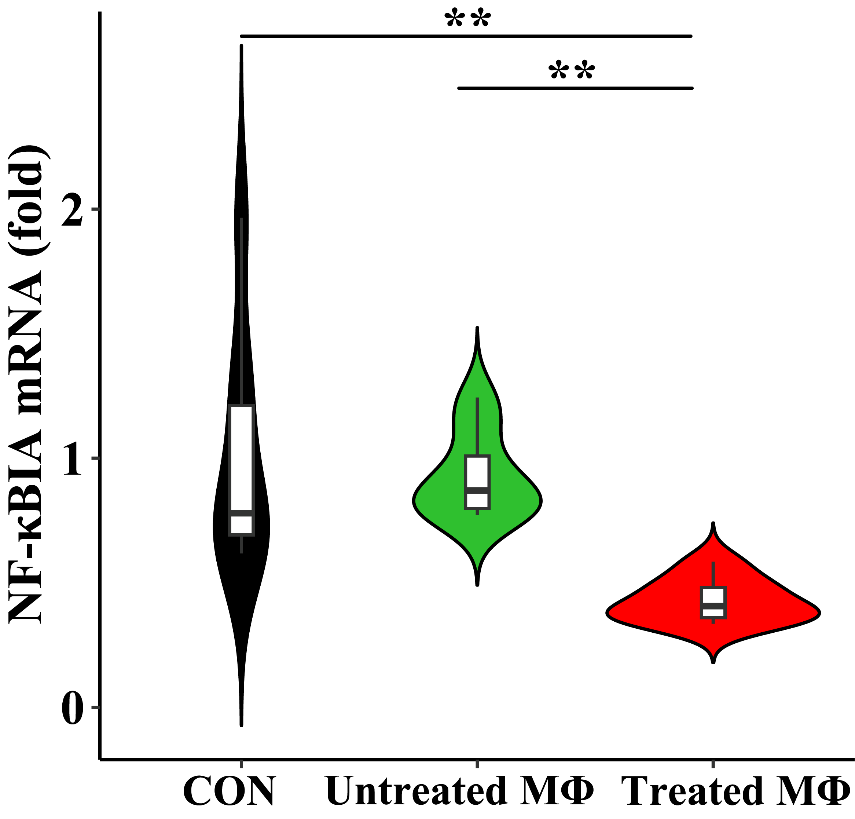


**Figure S8: NF-κBIA suppression in adoptive transfer model**

qPCR analysis of jejunal NF-κBIA expression. n = 8. Statistical differences were assessed using one-way ANOVA with Tukey's post hoc comparison, with data presented as mean values ± standard error of the mean. Significance thresholds: **P < 0.01.
